# The NeuroWES project: lessons learned from comprehensive phenotyping and genetic analysis of neurodevelopmental disorders over a decade

**DOI:** 10.1007/s00439-026-02843-4

**Published:** 2026-07-08

**Authors:** Simona Cardaropoli, Lisa Pavinato, Slavica Trajkova, Diana Carli, Verdiana Pullano, Flavia Palermo, Alessandro Mussa, Elisa Biamino, Vincenzo Antona, Andrea Zonta, Paola Dimartino, Mariia Zadorozhna, Alessandro Bruselles, Roberto Keller, Barbara Pasini, Enrico Grosso, Giorgia Mandrile, NeuroWES consortium, Andrea Angius, Carlo Arduino, Irene Bagnasco, Elga Fabia Belligni, Giovanni Birolo, Rachele Cantone, Silvia Carestiato, Chiara Davico, Manila Deiana, Eleonora Di Gregorio, Enza Ferrero, Giorgia Gai, Andrea Gazzin, Daniela Francesca Giachino, Chiara Giovenino, Andrea Guala, Antonella Maffè, Andrea Maschio, Matteo Massidda, Stefania Massuras, Alice Moroni, Valeria Giorgia Naretto, Alessandra Pelle, Anna Maria Pengo, Francesco Pintus, Aldamaria Puliti, Vincenzo Rallo, Evelise Riberi, Antonina Rinninella, Serena Rizzo, Federico Rondot, Paola Salmin, Fabio Sirchia, Lorena Sorasio, Benedetto Vitiello, Giulia Zacchetti, Joseph D. Buxbaum, Silvia De Rubeis, Tommaso Pippucci, Marco Tartaglia, Elisa Giorgio, Alfredo Brusco, Giovanni Battista Ferrero

**Affiliations:** 1 Department of Public Health and Pediatrics, University of Turin, 10126 Turin, Italy; 2 Institute for Oncology Research (IOR), Bellinzona Institutes of Science (BIOS+), Bellinzona, Switzerland; 3 Università della Svizzera Italiana, Lugano, Switzerland; 4 Department of Neuroscience “Rita Levi Montalcini”, University of Turin, Pzza Nizza 44, 10126 Turin, Italy; 5 Medical Genetics Unit, Città della Salute e della Scienza University Hospital, 10126 Turin, Italy; 6 Department of Medical Sciences, University of Turin, 10126 Turin, Italy; 7 Medical Genetics Unit and Thalassemia Center, San Luigi University Hospital, 10049 Orbassano, TO Italy; 8 Clinical Pediatrics Genetics Unit, Regina Margherita Children’s Hospital, Turin, Italy; 9 Department of Health Promotion, Mother and Child Care, Internal Medicine and Medical Specialties “Giuseppe D’Alessandro”, University of Palermo, Palermo, Italy; 10 Department of Molecular Medicine, University of Pavia, 27100 Pavia, Italy; 11 Department of Oncology and Molecular Medicine, Istituto Superiore di Sanità, 00161 Rome, Italy; 12 Adult Autism Centre DSM, ASL Città di Torino, 10138 Turin, Italy; 13 Department of Psychology, University of Torino, Turin, Italy; 14 Department of Genetic and Genomic Sciences, Icahn School of Medicine at Mount Sinai, New York, NY USA; 15 Department of Psychiatry, Icahn School of Medicine at Mount Sinai, New York, NY USA; 16 Seaver Autism Center for Research and Treatment, Icahn School of Medicine at Mount Sinai, New York, NY 10029 USA; 17 The Mindich Child Health and Development Institute, Icahn School of Medicine at Mount Sinai, New York, NY 10029 USA; 18 Icahn School of Medicine at Mount Sinai, Friedman Brain Institute, New York, NY 10029 USA; 19 The Alper Center for Neural Development and Regeneration, Icahn School of Medicine at Mount Sinai, New York, NY 10029 USA; 20 Medical Genetics Unit, IRCCS University Hospital, 40138 Bologna, Italy; 21 Molecular Genetics and Functional Genomics, Bambino Gesù Children’s Hospital, IRCCS, 00146 Rome, Italy; 22 Department of Clinical and Biological Sciences, University of Turin, Regione Gonzole 10, 10049 Orbassano, TO Italy; 23 Department of Neuroscience, Icahn School of Medicine at Mount Sinai, New York, USA

## Abstract

Exome sequencing (ES) has become a primary tool for diagnosing neurodevelopmental disorders (NDDs), yet the interpretation of genetic variants in large, heterogeneous cohorts presents significant challenges that automated pipelines often fail to resolve. This study showcases the complexities and novel findings derived from a decade-long analysis of 419 Italian NDD patient-parent trios. While ES established a molecular diagnosis in 36.5% of cases (53.8% in syndromic presentations), our investigation moves beyond diagnostic yield to highlight the critical value of manual curation integrated with deep phenotyping. We demonstrate how this rigorous approach uncovers complex disease mechanisms, revealing that variants initially misclassified as missense or stop-gain are, in fact, pathogenic splicing defects confirmed by functional analysis. Furthermore, we resolve the paradox of pathogenic truncating *PPM1D* variants in control databases by demonstrating their somatic mosaic nature, a crucial insight for population data interpretation. Our work also refines gene-disease correlations by challenging the established role of *MID2* in NDDs, providing further evidence for *DSCAM* as a high-confidence risk gene, and expanding the known phenotypic spectrum of disorders, such as a novel *GNAI2*-related syndrome lacking expected immune dysfunction. This study underscores that navigating the complexities of large NDD cohorts requires a detailed, expert-driven approach to not only enhance diagnostic yield but also to advance our fundamental understanding of rare disease genetics.

**Supplementary Information:**

The online version contains supplementary material available at 10.1007/s00439-026-02843-4.

## Introduction

Neurodevelopmental disorders (NDDs) are a broad group of conditions, including intellectual disability (ID), autism spectrum disorder (ASD), attention‐deficit/hyperactivity disorder (ADHD), epilepsy, global developmental delay (DD), and motor disorders. They typically manifest in childhood and are associated with the disruption of the tightly coordinated processes underlying brain development. Affecting ≥ 3% of children worldwide, NDDs have a strong genetic basis (Gilissen et al. 2014; Parenti et al. 2020).

Genetic variations, from rare highly penetrant mutations to common SNPs, contribute to their etiology, and there is significant genetic overlap between different NDDs (Satterstrom et al. 2020). Despite advances in sequencing, a substantial number of NDDs lack a clear genetic diagnosis.

Exome sequencing (ES) has proven to be ideal for detecting pathogenic coding variants, and it is now widely employed as a first-tier diagnostic tool, with a diagnostic yield of up to 31% for nonsyndromic NDDs and 53% for syndromic forms (Savatt and Myers 2021). Furthermore, ES studies have highlighted the phenomenon of genetic pleiotropy, wherein variants in the same gene can contribute to a range of distinct NDD phenotypes. This observation underscores the substantial genetic overlap between neuropsychiatric conditions within the broader NDD spectrum (Rees et al. 2021).

This study aims to present the complexities and novel findings derived from a decade-long genetic analysis of 419 Italian patient-parent trios with neurodevelopmental disorders (NDDs), going beyond the simple diagnostic yield of exome sequencing by demonstrating the critical value of manual curation integrated with deep phenotyping in analyzing this large, heterogeneous NDD cohort. We also highlight how this rigorous approach uncovers complex disease mechanisms, such as reclassifying variants initially misclassified as missense or stop-gain as pathogenic splicing defects. Finally, the study aims to refine gene-disease correlations by challenging the established role of *MID2* in NDDs, providing further evidence for *DSCAM* as a high-confidence risk gene, and expanding the known phenotypic spectrum of disorders, exemplified by a novel *GNAI2*-related syndrome lacking the expected immune dysfunction. This work ultimately underscores the necessity for a detailed, expert-driven strategy to enhance diagnosis and advance the fundamental understanding of rare disease genetics in NDDs.

## Materials and methods

### Subject recruitment and phenotypic characterization

Written informed consent was obtained from all participating families, and the study protocol was approved by the “Città della Salute e della Scienza” University Hospital Ethics Committee (CE n.0060884 del 12/06/2015), in accordance with the Declaration of Helsinki.

Most families recruited were trios (367; 87.6%), while the remaining consisted of quads (32; 7.6%) or larger family groups (7; 1.7%); additionally, 13 families (3.1%) had only one available parent (Table 1, Fig. S1a). Thirty-nine families had more than one affected sibling; for simplicity, we will refer to ES analysis in these families as trio-exome sequencing. Family history of NDDs was available for 121 cases (28.9%), and forty cases (9.5%) had at least one relative affected by a similar, but often milder, condition as the proband. Most subjects were children (68% in the 1–12 years age range) and the median age at the patient recruitment was 8.0 years (Table 1, Fig. S1b).

**Table 1:** Study cohort

Features	Solved	Unsolved	All	*p*-value^a^ (solved *vs* unsolved)
N (%)	153 (36.5)	265 (63.5)	419	
*Gender*				
Male	96 (33.1)	194 (66.9)	290	0.039
Female	57 (44.2)	72 (55.8)	129	
*Consanguineous parents*				
Yes	9 (34.6)	17 (65.4)	26	NS
No	144 (36.6)	249 (63.4)	393	
*Age* (years)				
0–1	4 (50.0)	4 (50.0)	8	NS
2–5	43 (33.6)	85 (66.4)	128	
6–12	52 (35.1)	96 (64.9)	148	
13–20	27 (42.2)	37 (57.8)	64	
Older than 20	27 (38.0)	44 (62.0)	71	
*Family structure*				
Large family	4 (57.1)	3 (42.9)	7	NS
Sibling couple	8 (25.0)	24 (75.0)	32	
Trio	134 (36.5)	233 (63.5)	367	
One parent missing	7 (53.8)	6 (46.2)	13	
*Clinical groups*				
ASD	7 (8.1)	79 (91.9)	86	< 0.001
ASD + DD/ID	22 (30.1)	51 (69.9)	73	
DD/ID	40 (38.5)	64 (61.5)	104	
Syndromic NDD	84 (53.8)	72 (46.2)	156	

^a^Pearson's Chi-squared test

Patients underwent deep phenotyping by clinical geneticists, with features translated into Human Phenotype Ontology (HPO) terms to facilitate genotype–phenotype matching. Across all clinical groups, males were predominant, comprising 69% (290 out of 419) of the subjects. This predominance was notably high in the ASD groups (with or without ID), with 80% being male (127 out of 159), resulting in a male-to-female ratio of approximately 4:1, which is consistent with the existing literature on ASD (Table 1, Fig. S2) (Dougherty et al. 2022).

### Exome sequencing, prioritization, and variant calling

DNA was extracted from total blood using the ReliaPrep Blood gDNA Miniprep kit (Promega, Madison, WI, USA) following manufacturer's protocol and quantified with a NanoDrop spectrophotometer (ThermoFisher Scientifics, Waltham, MA, USA). Chromosomal Microarray Analysis (CMA) was performed using a 60 K whole-genome oligonucleotide microarray (Agilent Technologies, Santa Clara, CA, USA).

Cases were enrolled in the Autism Sequencing Consortium (ASC) (https://asc.broadinstitute.org/) project and their gDNA sample was sequenced at the Broad Institute on Illumina HiSeq sequencers as previously described (Bauer et al. 2018; De Rubeis et al. 2014; Satterstrom et al. 2020).

Initial read alignment and variant calling were performed using an in-house pipeline based on GATK Best Practices. Reads were aligned to the UCSC GRCh37/hg19 assembly using BWA-MEM, and variants were called using GATK HaplotypeCaller v3.7 (Li and Durbin 2009). This approach reflected the diagnostic standards at the time of data acquisition. We used SnpEff v.4 (Cingolani et al. 2012) and dbNSFP v.3.5 (Liu et al. 2016) tools for variants functional annotation, including Combined Annotation Dependent Depletion (CADD) v.1.3 (Kircher et al. 2014), Mendelian Clinically Applicable Pathogenicity (M-CAP) v.1.0 (Jagadeesh et al. 2016) and Intervar v.0.1.6 for functional impact prediction (Li and Wang 2017). Thereby, the analysis was narrowed to variants affecting coding sequences or splice site regions. Variant filtering was conducted as a multi-stage process. Preliminary exclusions of common variations were performed using dbSNP150 and gnomAD ver.2.0.1. Subsequently, all candidate variants underwent a final re-annotation and frequency assessment using gnomADV4.1 non-UKB subset (updated April 19, 2024). Variants were discarded if they exhibited a Minor Allele Frequency (MAF) > 0.05 in the gnomAD v4.1 database or our internal exome database of ~ 2000 individuals (Chen et al. 2024), or sequencing coverage below 20x. Putative candidate variants were prioritized according to the predicted impact on protein-coding sequences, their presence in ClinVar (Landrum et al. 2025), zygosity and genetic mode of inheritance, clinical features, and the function of the encoded protein. Manual curation involved a literature-based assessment of each candidate gene’s relevance to the specific patient’s neurodevelopmental profile. For variants in genes not yet definitively linked to human disease, we evaluated tissue expression patterns (GTEx) and gene constraint scores (pLI, LOEUF) and initiated international collaboration through GeneMatcher. Evaluation of the clinical significance of the variants was performed following the consensus guidelines published by the American College of Medical Genetics and Genomics (ACMG; https://www.acmg.net), by using the Alamut Visual Plus (ver.1.9; Sophia Genetics, Lausanne, Switzerland) and multidisciplinary consensus meetings. Accordingly, variants were initially classified into five classes: “pathogenic” (P), “likely pathogenic” (LP), of “uncertain significance” (VUS), “likely benign” (LB) and “benign” (B), based on multiple lines of evidence. In most cases, P and LP variants were definitively considered as causative after segregation analysis when parental DNA was available and after evaluating the consistency with the clinical phenotype.

The previously unreported variants identified in this study were deposited in ClinVar (SUB15267594; Table S1).

### Splicing analysis

To confirm the predicted impact of splicing variants, we followed two alternative strategies.

In patient with *NFIB* variant, blood sample was drawn into PAXgene Blood RNA tube (PreAnalytiX, Hombrechtikon, Switzerland) and stored at − 80 °C. Total RNA was extracted using the PAXgene Blood RNA Kit, following the manufacturer’s instructions. Briefly, after centrifugation and removal of the supernatant, the cell pellet was lysed, followed by protein digestion and RNA binding to a silica membrane. Genomic DNA was removed via on-column DNase treatment, and purified RNA was eluted in RNase-free water. RNA quality and concentration were assessed using a Nanodrop spectrophotometer. Complementary DNA (cDNA) was generated using the M-MLV Reverse Transcriptase kit (Invitrogen, ThermoFisher Scientific). Primers were designed using Primer3plus platform (Table S2). cDNA was amplified with touchdown PCR using KAPA Taq PCR Kit (Roche Diagnostics, Basel, Switzerland). Amplimers were visualized into 2% TBE-agarose gel using ChemiDoc Imaging System (BioRad, Hercules, CA, USA). Bands were gel-excised, and the DNA was extracted by Monarch DNA Gel Extraction Kit (T1020S, NEB, Ipswich, MA, USA). Fragments were sequenced by the Sanger method.

For patient with *PUS3* variant, it was not possible to obtain a fresh sample for RNA extraction. To functionally validate these variants, minigene experiment was set up, as previously described (Mancini et al. 2012). Briefly, the genomic region containing the candidate splicing variant and adjacent exons was PCR-amplified from the patient using the KAPA2G Fast HotStart^®^ DNA Polymerase kit (Sigma-Aldrich) and custom primers (Table S2). The PCR products were then cloned into a pGEM^®^-T Easy Vector (Promega) and transformed into JM109 competent *E. coli* cells, following the manufacturer’s protocol. Plasmids containing either the wild-type (WT) or mutated (MUT) sequence were extracted using the ZymoPURE Plasmid MiniPrep kit (Zymo Research) and subsequently sub-cloned into the pSPL3 vector (Life Sciences-Invitrogen). The final pSPL3 plasmids (WT or MUT) were transfected into HeLa cells using Lipofectamine 2000 (Thermo Fisher Scientific). After 36 h, total RNA was extracted using the Direct-Zol RNA MiniPrep system (Zymo Research), cDNA was synthesized using the M-MLV Reverse Transcriptase kit (Invitrogen) and amplified with primers designed on the synthetic exons of the pSPL3 vector (SD6 and SA2; Table S2). The PCR products were purified using the DNA Clean & Concentrator kit (Zymo Research) and Sanger-sequenced with Big Dye Terminator chemistry (Thermo Fisher Scientific) to assess the splicing defect at the RNA level.

### Literature, database searches, and analytical tools

All the variants were described in accordance with the HGVS (https://www.hgvs.org/). We evaluated the clinical relevance of the identified variants and novel genes potentially associated with NDDs by consulting the following databases: ClinVar, Human Gene Mutation Database (HGMD), Online Mendelian Inheritance in Man (https://www.omim.org/), and DECIPHER (https://www.deciphergenomics.org/; v11.30). The GeneBe (https://genebe.net/; ver.0.0.1) (Stawiński and Płoski 2024) and MetaDome web tool (https://stuart.radboudumc.nl/metadome/) (Wiel et al. 2019) were employed to interpret variants further. Frequency of the variants in the population was assessed by gnomAD (ver.4.1). Specifically, SFARI (https://gene.sfari.org/) was used to obtain insights into genes implicated in autism susceptibility. PubMed and OMIM served as a resource for accessing existing literature on the function and role of encoded proteins or paralogous genes. Furthermore, the GTEx database (https://www.gtexportal.org/home/) was utilized to assess the expression patterns of candidate genes across different tissues, providing additional evidence for their potential involvement in NDDs. This comprehensive approach allowed for the filtering and prioritization of novel gene candidates for further analysis.

### Statistical analysis

Statistical analyses were conducted using R (v4.4.2) (R Foundation for Statistical Computing, Vienna, Austria). Pearson's Chi-squared test (with Yates' correction for 2 × 2 tables) was used for nominal variables. Comparisons between groups for continuous variables were performed using a two-tailed *t*-test. For non-normally distributed data, the non-parametric Mann–Whitney test was used for group comparisons. A p-value < 0.05 was considered statistically significant.

### Software availability

CADD (v.1.3): Combined Annotation Dependent Depletion for scoring the deleteriousness of variants. M-CAP (v.1.0): Mendelian Clinically Applicable Pathogenicity tool for pathogenicity prediction. Intervar (v.0.1.6): Clinical interpretation of genetic variants by the ACMG/AMP 2015 guidelines. Alamut Visual Plus (v.1.9): Used for evaluating the clinical significance of variants. Analytical Tools: GeneBe (v.0.0.1): Utilized for further variant interpretation. MetaDome (v.1.0.1): Web tool for protein-to-phenotype analysis.

## Results

### Cohort description and diagnostic yield

**Fig. 1:**
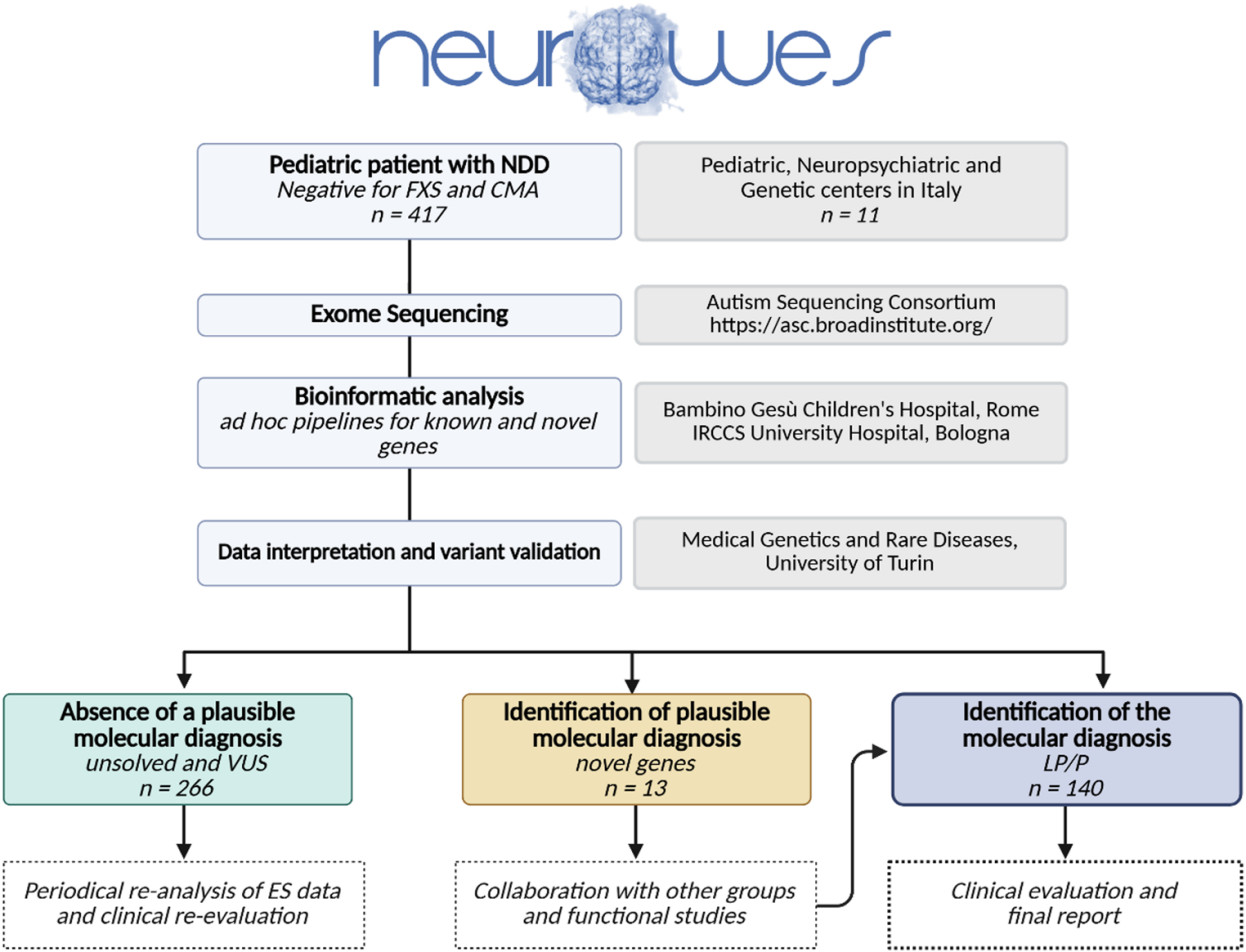
Workflow of the NeuroWES study. The study included 419 patients with NDDs (negative for fragile X syndrome (FXS) and Chromosomal Microarray Analysis (CMA) from pediatric, neuropsychiatric, and genetic centers in Piedmont and Sicily (Italy). The exome sequencing (ES) was performed by the Autism Sequencing Consortium (Satterstrom et al. 2020). Bioinformatic analysis utilized ad hoc pipelines to assess known and novel genes, conducted at Bambino Gesù Children's Hospital, Rome, and IRCCS University Hospital, Bologna. Variant interpretation and validation were performed by the Medical Genetics and Rare Diseases team at the University of Turin. Based on the results, patients were classified into three categories: (1) absence of a plausible molecular diagnosis (n = 266), which undergoes periodical re-analysis; (2) identification of a plausible molecular diagnosis involving novel genes (n = 13), requiring further collaborative work and functional studies; and (3) confirmed molecular diagnosis with likely pathogenic/pathogenic (LP/P) variants (n = 140), leading to clinical evaluation and final reporting. Created with BioRender.com [Created in BioRender. https://BioRender.com/6o90noo]

Between 2015 and 2019, we collected 419 unrelated individuals affected by neurodevelopmental disorders (NDDs), together with available family members, from multiple clinical centers across Italy (Fig. 1).

**Fig. 2:**
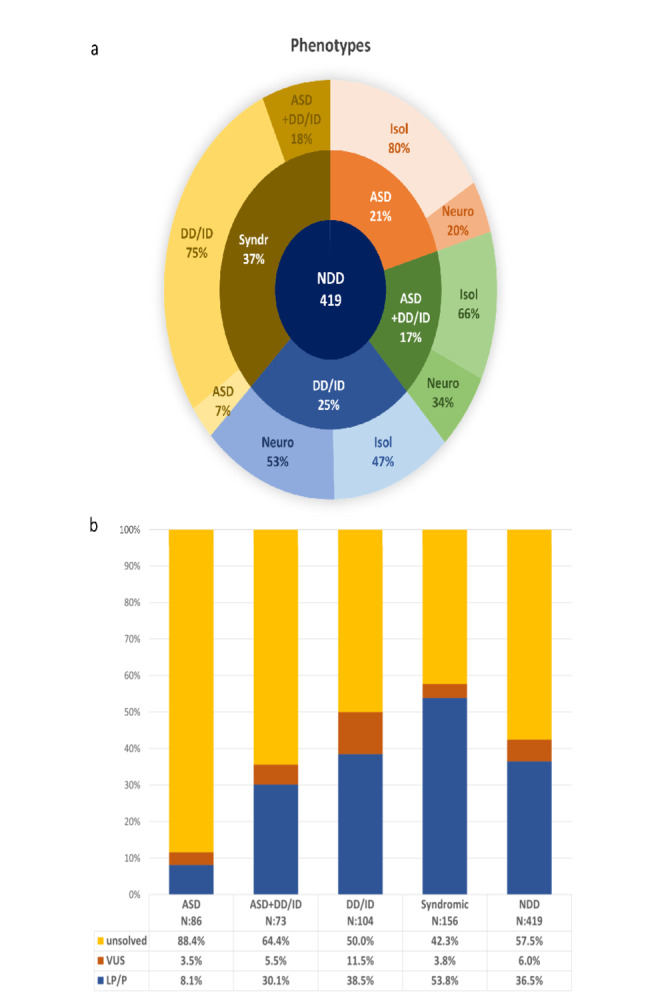
Phenotypes in NDD cases and genetic variation distribution of the NeuroWES cohort. **a** The inner circle represents the total cohort of NDDs (N = 419). The middle ring shows the distribution of specific phenotypes: ASD (20.5%), ASD + DD/ID (17.4%), DD/ID (24.8%), and syndromic NDDs (37.2%). The outer ring displays the breakdown of non-syndromic cases stratified into two subgroups: (i) a neurological subgroup, including patients with additional neurological features (e.g., micro/macrocephaly, epilepsy, or other neurological abnormalities), and (ii) an isolated subgroup, defined as individuals with ID and/or ASD in the absence of neurological signs or involvement of other organ systems; syndromic cases are further divided based on the occurrence of ASD, DD/ID, and ASD + DD/ID. **b** A stacked bar chart indicates the distribution of genetic variations, including unsolved, Variants of Unknown significance (VUS) and Pathogenic (P) or Likely pathogenic (LP). Variants are represented for different phenotypes such as ASD, ASD + ID, ID, syndromic disorders and the whole cohort (NDD)

All patients underwent a standardized pre-screening diagnostic workflow, which included clinical assessment, molecular testing for Fragile X syndrome (CGG expansion), as well as chromosomal microarray analysis (60 K, Agilent Technologies). Based on clinical presentation, the cohort was subdivided into four diagnostic groups: 86 cases with ASD (21%), 104 with DD/ID (25%), 73 with both conditions (17%) and 156 syndromic cases characterized by NDD associated with multisystemic involvement (37%) (Table 1, Fig. 2a).

The overall diagnostic yield, considering LP/P variants, was 36.5% (153/419). Diagnostic rates varied significantly across clinical categories: 8.1% in ASD only (7/86), 30.1% in ASD + DD/ID (22/73), 38.5% in DD/ID (40/104), and 53.8% in syndromic NDD (84/156) (Table 1, Fig. 2b).

### Inheritance patterns of pathogenic variants

**Fig. 3:**
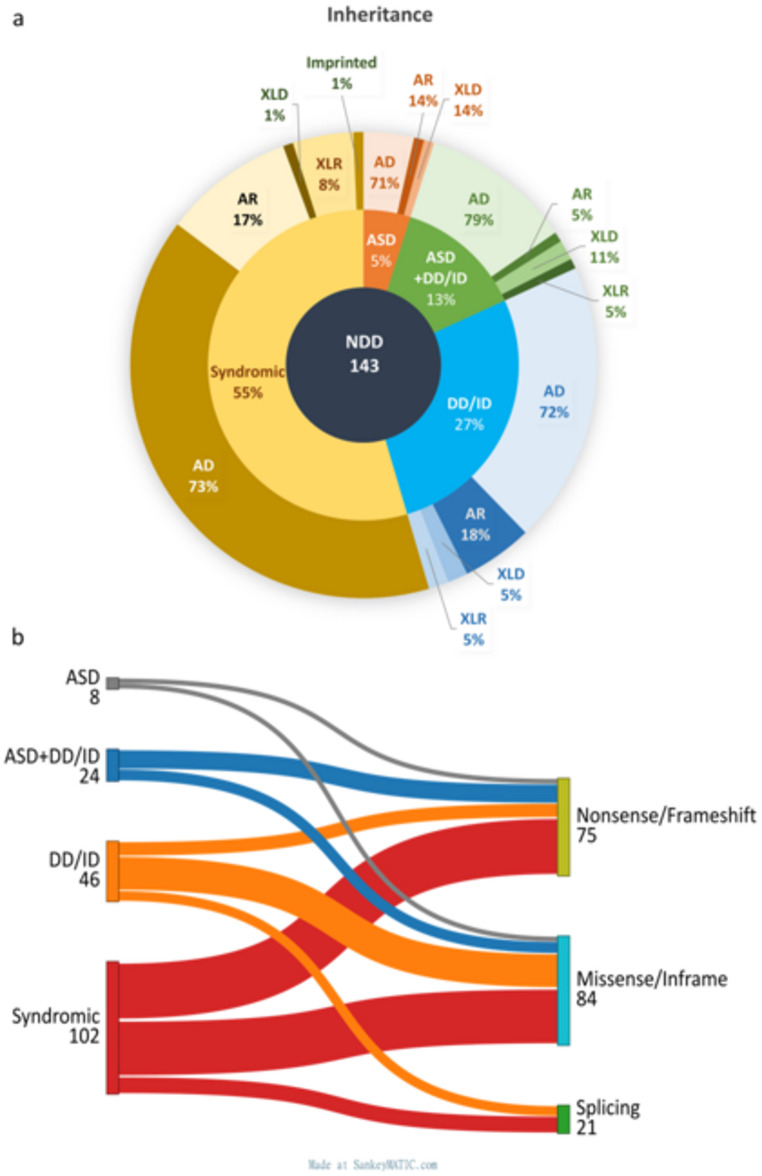
Inheritance patterns, main diagnosis and variant types identified in the NeuroWES solved cases. **a** The inner circle (NDD: 143) represents the total number of solved cases (excluding ten dual cases). The middle ring shows the contribution of each inheritance type to specific disorders, including syndromes, ASD, ASD + DD/ID, and DD/ID. The outer ring reflects neurodevelopmental disorders depicts the proportion of inheritance types: AD (Autosomal Dominant), AR (Autosomal Recessive), XLD (X-Linked Dominant), XLR (X-Linked Recessive), and Imprinted genes. **b** Gene variant effect and phenotypic Correlation. Sankey diagram that visualizes the relationship between NDD disorders and types of genetic variants: Nonsense/Frameshift, Missense/Inframe, and Splicing. It illustrates how these variant types SD, D/ID, and Syndromic NDD

Among the 143 genetically solved cases, the majority (105/143, 73.4%) showed an autosomal dominant (AD) mode of inheritance, excluding ten dual molecular diagnoses (Table 2, Fig. 3a). *De novo* origin was confirmed in 93% of AD cases (98/105)*,* constituting 68.5% of all solved cases. In four families, the LP/P variant was inherited from a mildly affected parent, suggesting reduced penetrance or variable expressivity (Table 2). These included: a male with complex phenotype including polydactyly, hypospadias, DD, and MRI anomalies, carrying a truncating *GLI2* variant inherited from a mother with mild polydactyly; a female with global DD, ID, seizures, and brain anomalies, carrying a *GLI2* splice site variant inherited from a father with epilepsy history; a girl with ID, microcephaly, and seizures, heterozygous for a *TLK2* nonsense variant inherited from a mother with mild ID; and a male with moderate ID and seizures, carrying a *CAPRIN1* nonsense variant inherited from a father with learning difficulties (Table S1).

**Table 2:** Inheritance

	Cases without dual (N = 143)^a^
Autosomal dominant	105 (73.4%)
*De novo*	98
Inherited	4
Unknown	3
Autosomal recessive	22 (15.4%)
Homozygous	11
Compound heterozygous	11
X-linked	15 (10.5%)
Dominant	6
Recessive	9
Imprinted	1 (0.7%)

^a^Ten cases with a "dual" diagnosis or molecular finding were excluded from the inheritance analysis

Autosomal Recessive (AR) inheritance was observed in 22 patients (15.4% of solved cases), often involving compound heterozygosity or homozygosity. Homozygous variants were found in 11 cases; 4 of these families reported consanguinity. AR inheritance was rare in ASD + ID cases (5%, 1/19) (Table 2, Fig. 3a).

X-linked inheritance (XL) accounted for 15 cases (10.5%). This included six X-linked dominant cases (4.2%), four with *de novo* variants (in *DDX3X, HNRNPH2, IQSEC2, KDM6A*) and two inherited (*ACSL4, FMR1*). Nine cases showed X-linked recessive inheritance (6.3%), mostly maternally inherited, except for two with *de novo CNKSR2* and *SLC6A8* variants (Fig. 3a, Table 2 and S1).

An imprinted inheritance pattern was identified in a single case involved a paternally inherited frameshift variant in *MAGEL2*, consistent with Schaaf-Yang syndrome.

### Variant type spectrum

Among 180 LP/P variants identified, missense/in-frame variants were the most common (84, 46.7%), followed by nonsense/frameshift (75, 41.7%), and splicing site variants (21, 11.7%)(Fig. 3b). Of these, 28 variants were previously reported, mainly *de novo* autosomal dominant cases. Two were X-linked dominant: *FMR1* p.(Arg442Gln) and *HNRNPH2* p.(Arg206Gln) (Table S3) (Bain et al. 2016; Harmsen et al. 2019; Zeidler et al. 2021). The *FMR1* case was described in detail elsewhere (Mangano et al. 2022).

### Dual molecular diagnoses

Ten cases had likely or possible dual diagnosis (Table S4). Only three involved two LP/P variants (*TLK2* + *DYNC1H1, RAB3GAP1* + *PAH*, and *NOTCH2* + *ASXL1*). In most dual diagnosis cases, at least one variant was classified as a VUS. Clinically, eight of these ten cases showed a single blended phenotype, while two patients (*RAB3GAP1* + *PAH* and *NOTCH2* + *ASXL1*) presented with distinct syndromic components (Table S4).

### Gene discovery and expanding disease phenotypes

#### Novel gene-phenotype associations

During the project, we identified 13 genes not previously associated with NDDs at the time of analysis. Three of them (*KCNK18*, *RPH3A*, *ZMYM3*) were later validated through follow-up studies (Table S5) (Hiatt et al. 2023; Pavinato et al. 2021a, 2023). Collaborative efforts expanded the clinical spectrum of known disease genes such as *EP300* exon 20 skipping (Pavinato et al. 2025), *PI4KA* variations (Neurodevelopmental disorder with spasticity, hypomyelinating leukodystrophy, and brain abnormalities; NEDSPLB OMIM # 616531), and Skraban-Deardorff syndrome (*WDR26*) (Pavinato et al. 2021b; Verdura et al. 2021).

#### *GNAI2* and novel phenotype

**Fig. 4:**
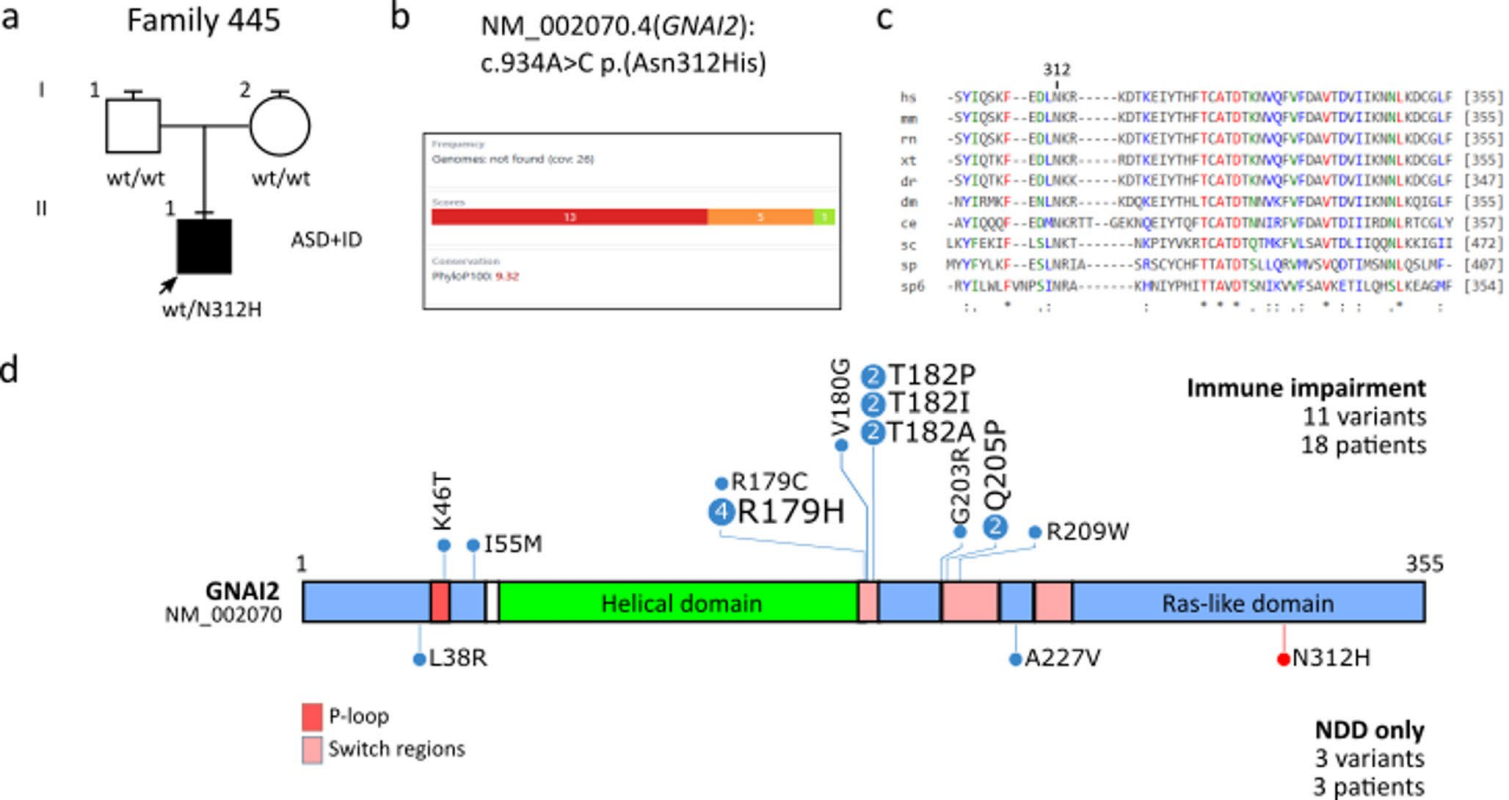
*GNAI2* missense variant. **a** The pedigree illustrates that the NM_002070.4(*GNAI2*):c.934A > C p.(Asn312His) variant is *de novo*. **b** *In silico* tools predict a pathogenic outcome for this variant, as summarized on the GeneBe website, suggesting a possible novel phenotype associated with *GNAI2* (Stawiński and Płoski 2024). The Asn312 residue is highly conserved among species (Wang et al. 2017) **c** The variant is absent in controls (gnomAD ver.4.1), and in ClinVar. **d** created with St. Jude Cloud PeCan (https://pecan.stjude.cloud/variants/proteinpaint), illustrates the location of this and known variants on the GNAI2 protein: the variants above the protein are associated with immune impairment (Ham et al. 2024) while those below the protein are linked exclusively to NDDs (Ham et al. 2024; Hamada et al. 2017). The p.(Asn312His) variant identified in this study is shown in red

A proband (family 445) carried a rare *GNAI2* missense variant NM_002070.4: c.934A > C p.(Asn312His) (Fig. 4a). Activating G_αi2_- mutations are associated with a complex clinical presentation that includes abnormal development characterized by intrauterine growth retardation, dysmorphism, bone dysostosis, neuroanatomical abnormalities, birth defects, and variable immune dysfunction (Ham et al. 2024). Unlike previously reported cases with significant immune dysfunction, this proband had autistic behavior, ADHD, mild intellectual disability, and delayed speech and language development, but no evidence of immune impairment. Two additional cases from independent studies carrying similar *GNAI2* variants recently reported also lacked immune involvement (Ham et al. 2024; Hamada et al. 2017) (Fig. 4d). The variant pathogenicity was supported by *in silico* prediction and ACMG criteria (Fig. 4b). Interestingly, the amino acid affected in the RAS domain is highly conserved, and the variant was private (Fig. 4c, d). It is currently unclear which pathogenic mechanism could underly this variant phenotype.

### Disease mechanisms and variant reinterpretation

#### *DSCAM* haploinsufficiency in NDDs

In family 87, we identified a *de novo* loss-of-function (LoF) variant in the *DSCAM* gene (Figure S3a). *DSCAM* (Down Syndrome Cell Adhesion Molecule), located on chromosome 21q22, encodes a cell adhesion molecule involved in axon guidance, dendritic self-avoidance, and synaptic targeting, which are crucial processes in early brain development. This gene is highly constrained for LoF variants (pLI = 1; LOEUF = 0.11), suggesting strong purifying selection against heterozygous disruptive alleles in the general population.

Although *DSCAM* has been previously implicated in autism spectrum disorder (ASD) in both human studies and animal models (Chen et al. 2022; De Rubeis et al. 2014; Iossifov et al. 2014; Lim et al. 2021; Monies et al. 2017; Satterstrom et al. 2020; Wang et al. 2016), a definitive syndromic characterization has not yet been established. To further investigate its pathogenic potential, we reviewed publicly available variant databases (DECIPHER, ClinVar, PubMed) and identified 19 individuals carrying monoallelic, likely gene-disrupting variants (including nonsense, canonical splice-site, and frameshift mutations). Despite limited clinical annotation, the majority of these individuals present with neurodevelopmental phenotypes, with ASD being the most consistently reported feature, often accompanied by intellectual disability (ID) of variable severity (Fig. S3b).

#### Splicing aberrations in initially misclassified variants

**Fig. 5:**
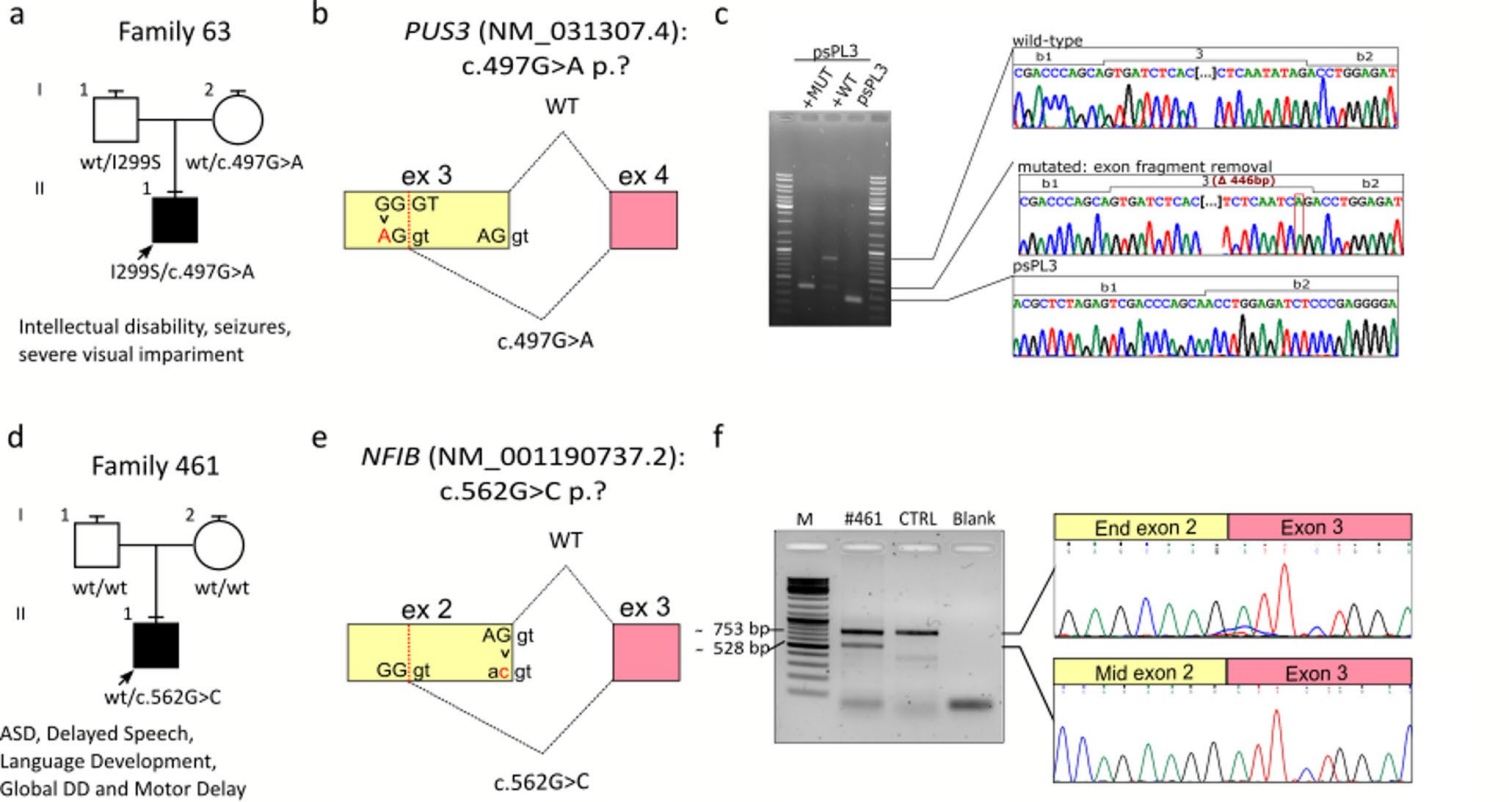
Splicing variants. **a** The pedigree of family 63 shows that the proband has inherited a pathogenic *PUS3* variant from each parent. **b** The variant NM_031307.4(*PUS3*): c.497G > A affects the 119 base of exon 3 and leads to an imbalanced expression of alternative transcripts due to a cryptic donor splice site gain. **c** A gel electrophoresis image shows products from a minigene splicing assay: wild-type, mutant, and vector controls. Sanger sequencing traces confirm that the variant causes exon fragment removal (446 bp), producing an abnormally shortened transcript compared to the wild-type construct. **d** In family 461, the pedigree reveals a *de novo* variant in the *NFIB* gene present in the proband. **e** The specific variant, NM_001190737.2(*NFIB*): c.562G > C, affects the last base of exon 2. This alteration disrupts normal splicing, leading to aberrant transcripts that could affect the gene's function. **f** Presents the results of gel electrophoresis performed on cDNA from both the patient and a control, using primers located in exon 1 and exon 4 (Table S2). The proband (#461) exhibits a distinct band at 528 base pairs, indicative of a partial loss of 225 base pairs from exon 2, as confirmed by the chromatograms on the right. This splice alteration results in an in-frame spliced mRNA variant compared to the wild-type sequence

To bridge the gap between bioinformatic prediction and molecular diagnosis, we performed functional validation on candidate splicing variants in *PUS3, NFIB* and *ARID1B*. For the *PUS3* variant in Family 63 (c.497G > A), initially annotated as a missense change [p.(Arg166Gln)], a minigene assay using the pSPL3 vector system demonstrated that the mutation triggers the activation of a cryptic splice site. This results in an out-of-frame deletion of exon 3, which is predicted to introduce a premature termination codon and subsequently trigger nonsense-mediated decay (Fig. 5a–c). In family 461, cDNA analysis of the *NFIB* variant c.628G > C, initially predicted as p.(Asp188His), confirmed that the substitution disrupts the canonical splice donor site, causing in-frame skipping of exon 2 (Fig. 5d–g).

Finally, we re-evaluated a variant in *ARID1B* variant (c.2480C > T; Family 98), which was initially prioritized as a missense change [p.(Ala827Val)]. While its pathogenicity was independently supported by a diagnostic episignature, *in silico* tools and recent literature (Bosch et al. 2024; Trajkova et al. 2024) strongly suggest an additional or primary effect on mRNA splicing.

#### Rare missense variants in haploinsufficient genes

We identified ultra-rare missense variants in *RBM10* and *AHDC1*, both of which are intolerant to LoF. In the X-linked gene *RBM10*, typically implicated in NDD risk via loss-of-function truncating variants, we found the p.(Arg766Cys) missense change. This variant is in the C-terminal Zinc Finger domain (C2H2-type ZF), which is one of the key functional domains of the RBM10 protein. The change hit a very highly conserved aminoacid in vertebrates (highly intolerant change, Metadome website) within a highly conserved protein region (a.a. 692–798). Computational scores are concordant to determine pathogenicity with MetaRNN supporting a deleterious effect (0.888) (Li et al. 2022) (Li et al. 2022). This suggests the critical amino acid alteration may represent a rare LoF *RBM10* missense change (Johnston et al. 2010; Niceta et al. 2019) (Fig. S5).

In family 255, we found a rare *AHDC1* missense variant p.(Pro1009Ala), a gene associated with Xia-Gibbs syndrome (syndromic ID) predominantly through *de novo* truncating variants. The clinical features observed in our patient, including DD/ID, hypotonia, seizures, sleep apnea, hypoplasia of the corpus callosum, atrial septal defect, and feeding difficulties, are consistent with the phenotype spectrum reported for individuals with *AHDC1* missense variant. This variant affects a highly conserved amino acid and is predicted to be structurally damaging by bioinformatic software (Fig. S6) (Khayat et al. 2021).

#### Reevaluation of *MID2*

In Family 309, our patient had a *de novo* likely pathogenic variant in *GNB1* (c.833_835del, p.(Phe278del)), a gene associated with Intellectual Developmental Disorder, Autosomal Dominant 42 (OMIM# 616973), fully explaining the phenotype (Figure S7a); in this proband, we also identified a *de* *novo* frameshift variant in *MID2* (NM_012216.4:c.2070del, p.(Phe691Leufs*8)). We noted that *MID2,* proposed as an X-linked ID gene, currently holds a 'provisional' status in the OMIM database (OMIM# 300928). Our re-analysis of the gene leads us to suggest caution regarding this association. A primary concern involves the single existing report (Geetha et al. 2014), where our re-evaluation of the provided linkage data suggests an incorrect assignment of the minimal linkage region. The original researchers erroneously excluded the uncertain regions identified by linkage between DXS991-DXS986 and DXS1001-DXS1047. This miscalculation potentially overlooked a region of over 33 Mb which contains several known ID-associated genes already associated with ID, including *ARHGEF9, ATRX, GRIA3, TAF1,* and *THOC2*. Furthermore, the p.(Arg347Gln) variant found in their family is currently reported in ten male subjects in the gnomAD database (version 4.1), an allele frequency that exceeds the expected threshold for a rare Mendelian neurodevelopmental disorder. Finally, bioinformatics predictions do not support a relevant functional role for the *MID2* p.(Arg347Gln) substitution, further weakening its potential pathogenicity (Fig. S7c–e).

#### *PPM1D* nonsense variants: mosaicism insight into Jansen-de Vries syndrome

**Fig. 6:**
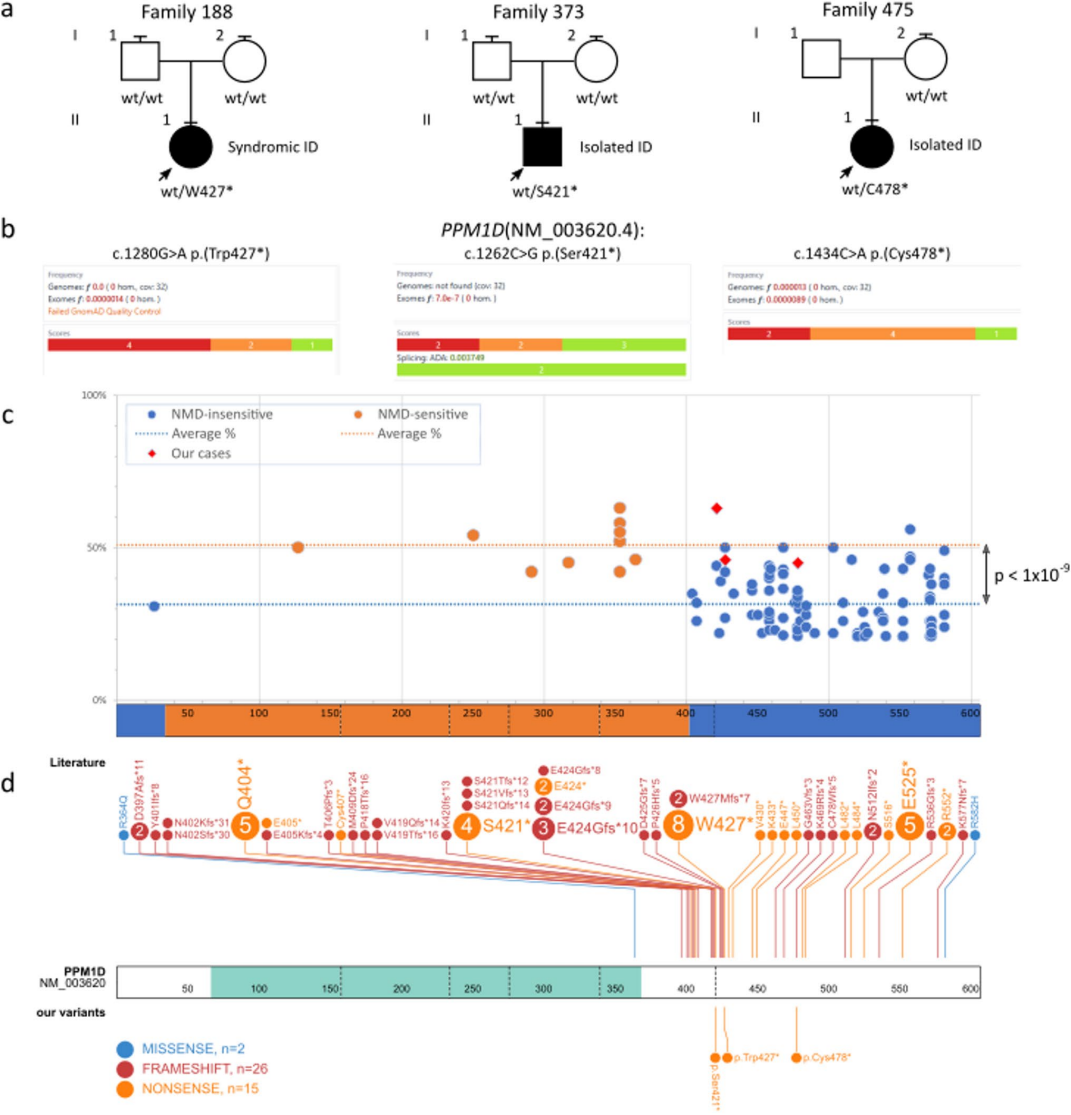
*PPM1D* nonsense variants. **a** We identified three unrelated cases with *de novo* stop gain variants in *PPM1D*(NM_003620.4). Among these, family 188 has been previously reported by (Wojcik et al. 2023). **b** All identified variants are predicted to be pathogenic (GeneBe website) (Stawiński and Płoski 2024). **c** Distribution of mutant allele ratios for stop gain variants within *PPM1D* in controls from the gnomAD database (gnomADV4.1). The data show that these variants have an approximate 50% allele ratio in regions sensitive to nonsense-mediated decay (NMD) (orange bar and dots). Conversely, in NMD-insensitive regions (blue bars and dots), the average is approximately 31%. The difference in allelic ratios between NMD-sensitive and NMD-insensitive regions is statistically significant (p value < 1 × 10^− 9^). Our variants, indicated by red diamonds, are also present in the gnomAD v4.1 (gnomADV4.1): p.(Trp427*) and p.(Ser421*) variants were each observed once (allele fraction 27% and 44%), while the p.(Cys478*) variant was detected 10 times with variant (allele fraction of 21–36%). Panel **d** created with St. Jude Cloud PeCan (https://pecan.stjude.cloud/variants/proteinpaint), illustrates the location of all causative *PPM1D* variants described in both the literature and this manuscript (Cunningham et al. 2024; Li et al. 2020; Tsai et al. 2022; Wojcik et al. 2023)

We identified three cases (Families 188, 373, 475) with *de novo* stop-gain variants in the last exon of the *PPM1D* gene (Fig. 6a). Variants of this type escape nonsense-mediated decay (NMD), are the established cause of Jansen-de Vries syndrome (JDVS), an autosomal dominant NDD matching our patients' clinical profiles (Jansen et al. 2017; Wojcik et al. 2023)(Fig. 6a). However, we encountered a significant paradox: our variants were also present in unaffected individuals within the gnomAD control database (gnomADV4.1) (Fig. 6b). To resolve this contradiction, we hypothesized that the presence of these variants in the general population could be explained by somatic mosaicism. To test this, we analysed the allele balance of all nonsense variants across the *PPM1D* gene in the gnomAD database. This investigation revealed a striking pattern: variants located in the NMD-ineffective region (the beginning of exon 1, the end of exon 5 and all of exon 6) were consistently in a mosaic state. Conversely, variants in the NMD-effective region (most of exon 1 through most of exon 5) showed a normal, balanced allele distribution. This difference was highly statistically significant (p < 1 × 10^− 9^), confirming that the pathogenic *PPM1D* variants found in the control population are not constitutional but are instead mosaic (Fig. 6c). This finding resolves the initial paradox and highlights that haploinsufficiency of the *PPM1D* is likely not associated with disease, and that pathogenic variants are only in the NMD-incompetent region (Fig. 6d).

#### Clinical actionability

We found that 17 of the identified genes had actionable implications, meaning the specific genetic diagnosis can inform therapeutic choices, dictate necessary clinical surveillance, or influence overall patient care protocols (Table 3). Most of these genes were connected to cancer, suggesting that these patients could benefit from oncologic surveillance; others were associated with treatments reported in databases such as Clin Gen (https://clinicalgenome.org/), ClinPGx (https://www.clinpgx.org/), and Rx-genes (https://www.rx-genes.com/) that could ameliorate clinical features.

**Table 3:** Clinically actionable genes

Gene	Variant	Disease	Outcome	Treatment/Intervention
*BSCL2*	NM_001122955.4: c.1076dup; p.(Glu360*)	Encephalopathy, progressive, with or without lipodystrophy	Lipodystrophy*	Metreleptin is indicated as an adjunct to diet as a replacement therapy to treat the complications of leptin deficiency^a,b^
*CDK13*	NM_003718.5: c.2506A > G; p.(Arg836Gly)	Congenital heart defects, dysmorphic facial features, and intellectual developmental disorder	Cardiovascolar disease, Seizure Growth delays or hormone imbalances	Cardiac surveillance^c^ Seizure monitoring Endocrine and Growth monitoring
*CHD7*	NM_017780.4: c.6194G > A; p.(Arg2065His)	CHARGE syndrome	Immunodeficiency	Immunologic surveillance^c^ Hematopoietic stem cell transplantation (HSCT)—bone marrow transplant^a^
*COL4A1*	NM_001845.6: c.2528G > T; p.(Gly843Val)	Brain small vessel disease with or without ocular anomalies	Hypertension Brain hemorrhage risk* Kidney, eye, and muscle complications*	Blood Pressure Control^c^ Avoidance of Anticoagulants & Antiplatelets Minimize Head Trauma & Strenuous Activities Regular Brain Imaging (MRI, CT Scan) Routine Eye Exams Regular Kidney Function Tests Hydration & Kidney Protection Muscle & Movement Support
*CREBBP*	NM_004380.3: c.3779 + 1G > A; p.?	Rubinstein-Taybi syndrome 1	Cardiovascolar disease*, Tumors	^c^Cardiac surveillance Surveillance to detect tumors and guide treatment
*GLA*	NM_000169.3: c.644A > G; p.(Asn215Ser)	Fabry Disease	Cardiovascular disease, Cerebrovascular events, End-stage renal disease	Enzyme replacement therapy (ERT) with agalsidase alpha or beta^c^ Migalastat is indicated for long-term treatment of adults and adolescents aged 16 years and older with a confirmed diagnosis of Fabry disease and who have an amenable mutation^b^
*KCNT1*	NM_020822.3: c.2459C > T; p.(Pro820Leu)	Epileptic encephalopathy, early infantile, 14; Epilepsy nocturnal frontal lobe, 5	Seizure	quinidine for gain of function variants^a^
*KMT2A*	NM_001197104.2:c.3615C > A; p.(Tyr1205*)	Wiedemann-Steiner syndrome	Leukemia	Immunologic surveillance^c^
*NF1*	NM_001042492.3:c.3497-1G > C; p.?	Neurofibromatosis type 1	Malignant peripheral nerve sheath tumors Breast Cancer Plexiform neurofibromas	Consultation with a provider experienced with NF1 for educational purposes^c^ Surveillance Selumetinib^a^
*PAH*	NM_000277.3:c.754C > T; p.(Arg252Trp)	Phenylketonuria (PKU)	PAH deficiency-related morbidity from elevated blood phenylalanine levels* Adverse pregnancy outcomes	Evaluation and management by specialists to achieve target phenylalanine levels with dietary and/or pharmacologic therapies^c^ Low protein diet, pegvaliase, tetrahydrobiopterin^a^ Pre-conception and pregnancy management by specialists to achieve target phenylalanine levels with dietary and/or pharmacologic therapies^c^
*PTEN*	NM_000314.8:c.449A > G; p.(Glu150Gly)	PTEN Hamartoma Tumor Syndrome—Cowden Syndrome	Morbidity due to thyroid disease Morbidity due to breast cancer	Surveillance to detect thyroid lesions and guide treatment^c^ Surveillance to detect breast tumors and guide treatment
*PTEN*	NM_000314.8:c.243 T > G; p.(Phe81Leu)	PTEN Hamartoma Tumor Syndrome—Cowden Syndrome	Morbidity due to thyroid disease Morbidity due to breast cancer	Surveillance to detect thyroid lesions and guide treatment^c^ Surveillance to detect breast tumors and guide treatment
*PTPN11*	NM_002834.5:c.209A > G; p.(Lys70Arg)	Noonan syndrome	Cardiac manifestations*	Cardiac surveillance^c^ Cardiac surveillance with modification of physical activities as appropriate
*RPE65*	NM_000329.3: c.1206G > A; p.Trp402* NM_000329.3:c.1207_1210dup; p.Glu404Alafs*4	Biallelic RPE65 Mutation-Associated Retinal Dystrophy	*Progression of visual impairment	Gene therapy (Luxturna), limited to clinically affected individuals with viable retinal cells^a,c^
*SCN2A*	NM_001040142.2:c.3839 T > C; p.(Leu1280Pro)	Developmental and epileptic encephalopathy 11	*Seizure	^a^Phenytoin; high dose carbamazepine
*SCN2A*	NM_001040142.2:c.1871dup; p.(Asn624Lysfs*23)	Developmental and epileptic encephalopathy 11	Seizure	^a^Phenytoin; high dose carbamazepine
*SCN8A*	NM_001330260.2:c.2734 T > G; p.(Cys912Gly)	Developmental and epileptic encephalopathy 13	Seizure	Phenytoin; high dose carbamazepine^a^
*SLC13A5*	NM_177550.5:c.655G > A; p.(Gly219Arg)	Developmental and epileptic encephalopathy 25, with amelogenesis imperfecta	Seizure*	Ketogenic diet, stiripentol^a^
*SLC6A8*	NM_005629.4:c.1428C > G; p.(Tyr476*)	Cerebral creatine deficiency syndromes	Morbidity due to creatine transporter deficiency (males only)*	Referral to specialist to guide creatine, arginine, and glycine supplementation^c^

From ^a^ https://www.rx-genes.com, and/or from ^b^ clinpgx.org or from ^c^ https://clinicalgenome.org

*Observed in the proband

## Discussion

Over a decade, we leveraged an interdisciplinary team comprising clinicians, medical geneticists, bioinformaticians, and molecular geneticists to analyze a cohort of 419 NDD cases using trio-ES. Unlike many other studies that rely heavily on automated data processing, our research incorporated a comprehensive, manual review of genetic variants paralleling the deep phenotyping approach of the clinicians. This manual and collaborative effort aimed to ensure the highest accuracy in variant interpretation and provided detailed, nuanced insights into both common and unique phenotypic presentations associated with the identified genetic variants.

We found a high diagnostic second-tier yield of 36.5%, a statistic that resonates with similar studies in the field (Nakhleh Francis et al. 2023). Diagnostic rates varied significantly across clinical subgroups, with syndromic presentations showing a markedly higher yield (53.8%) compared to isolated ASD (8.1%).

Despite the high diagnostic yield achieved through integrated manual curation and deep phenotyping, our study has inherent limitations. Our primary genetic screening relied on short-read exome sequencing (ES), which is highly effective for detecting single-nucleotide variants (SNVs) and small insertions/deletions (indels) in coding regions but possesses reduced sensitivity for structural variants (SVs) and copy number variants (CNVs). While all patients were pre-screened with chromosomal microarrays (CMA), which effectively detects large-scale imbalances, smaller intragenic CNVs or complex rearrangements often remain undetected by both CMA and standard ES pipelines. Furthermore, our approach does not capture variation in non-coding regulatory elements or deep intronic regions that may influence gene expression or splicing.

AD inheritance surfaced as the predominant mode in our cohort, aligned with other exome studies, indicating its central role in NDDs. Notably, 93% of AD cases arose from *de novo* variants, underscoring the recurrence of spontaneous genomic alterations in neurological impairments (Fu et al. 2022; Satterstrom et al. 2020). Interestingly, only a few AD variants were inherited from mildly affected parents, reflecting reduced penetrance or variable expressivity, common features in clinical genetics.

In our cohort of solved cases, AR and XL inheritance patterns were less prevalent, observed in 15.4% and 10.5% of families, respectively. Interestingly, even within the 26 consanguineous families, only nine were solved and six carried homozygous AR variants. This relatively low yield of recessive causes likely reflects several factors. First, the low rate of parental consanguinity (6.2%) in our population naturally shifts the genetic architecture toward *de novo* dominant events (Srivastava et al. 2019). However, the remaining ‘unsolved’ recessive families may harbor variants in novel genetic loci that currently lack definitive clinical association (Bakur et al. 2025). Furthermore, the possibility of oligogenic inheritance cannot be ignored. The identification of ten dual molecular diagnoses in our study suggests that the genetic etiology in some NDD cases is more complex than a single-gene model. Moreover, unusual inheritance patterns, such as the imprinted inheritance of *MAGEL2* in Schaaf-Yang syndrome, add intriguing layers to our understanding (Schaaf et al. 2013). Finally, among the cases that were successfully solved, 6.5% (10 out of 153) were classified as dual cases. This percentage closely matches previously reported frequencies (Posey et al. 2017; Smith et al. 2019). In all but one of these dual cases, the phenotype involved overlapping diagnoses, indicating a heightened level of complexity in identifying them within the studied cases. This overlap suggests an increased challenge in diagnosis and highlights the need for careful consideration and differentiated approaches when evaluating such cases.

We observed that the diagnostic rate in families with multiple affected siblings was comparable to that of isolated cases, with rates of 30.8% (12 out of 39) and 37.1% (141 out of 380), respectively. Although we initially anticipated that familial cases might exhibit a higher diagnostic rate, potentially due to the involvement of autosomal recessive and X-linked genes, we also recognized that autistic children from multiplex families are reported to have an additive and complex genetic risk architecture for ASD, involving both rare and common genetic variations (Cirnigliaro et al. 2023). This complexity in genetic risk architecture could similarly apply to other NDDs. The identification of the underlying genetic cause holds particular clinical value when the involved gene is actionable. In our cohort, 19 of the 153 diagnosed cases (12.4%) carried medically actionable findings, highlighting the clinical utility of exome sequencing beyond diagnosis.

We identified 25 cases with VUS representing the likely event underlying the condition, accounting for 6% of our cohort. Almost all these VUS were missense changes, underscoring the challenges in classifying such variants. These findings warrant further investigation through functional studies and family segregation analyses. Future insights may help elucidate their potential role in disease pathology.

Despite having more males in our global cohort, we found a higher percentage of variants in NDD females vs. males (44.2% vs. 33.1%, *p*-value = 0.039; Odds ratio 1.598; 95% CI 1.021 to 2.499) (Table 1, Fig. S2b) (Antaki et al. 2022; Jacquemont et al. 2014; Turner and Eichler 2019). Although the "female protective model" suggests that females require a higher "mutational burden" for the clinical manifestation of NDDs, recent evidence challenges this view (Dougherty et al. 2022). It has been shown that autosomal rare and damaging coding variants confer similar liability for ASD in both females and males, allowing them to reach the diagnostic threshold. The differences we observed, combined with recent results from the literature, might align with the Differing Liability Distribution Models, which propose influences from sex chromosomes, epigenetic differences, placental biology, and hormonal (Dougherty et al. 2022; Koko et al. 2025).

A key finding of NeuroWES study was the identification of novel genes associated with NDDs, described in detail in other works, among which *KCNK18, RPH3A,* and *ZMYM3* (Hiatt et al. 2023; Pavinato et al. 2021a, 2023). This discovery expands the known genetic landscape of NDDs and contributed to the diagnostic yield achieved in our cohort. This is consistent with ongoing genomic research that continually identifies previously unrecognized genetic contributors to disease (Lek et al. 2016). Among these genes, *KCNK18* was found in a family with three affected siblings who presented with ID, epilepsy, and ASD (Pavinato et al. 2021a). The association of this gene with the disease was recently reinforced by a report of a six-year-old male from an Indian family. This individual exhibited mild motor delay, speech delay, moderate ID, ASD, and epilepsy with febrile seizures plus and a homozygous stop-gain variant, p.(Arg167*) (Majethia et al. 2023).

New cases associated with genes *CAPRIN1, KCNK2*, and *MYH10* largely echoed phenotypic descriptions in existing studies. On the other hand, the clinical spectrum of *GNAI2*-related disorders appears increasingly pleiotropic. While some variants cause a multisystemic syndrome with severe immune and skeletal involvement, our identification of the p.(Asn312His) variant adds to a growing subset of cases characterized by neurodevelopmental delay and growth restriction in the absence of immune dysfunction. This phenotypic divergence likely reflects the complex role of Gαi2 as a central transducer in multiple signaling pathways (Ham et al. 2024).

The functional characterization of variants allows a deeper insight into pathogenic mechanisms. For instance, missense variants, like those in *RBM10* and *AHDC1*, highlighted atypical scenarios where amino acid changes might cause LoF effects, expanding upon traditional genotype–phenotype correlations (O'Roak et al. 2012). Similarly, splicing alterations in genes previously classified as missense or nonsense reveal the complexity and depth of interpreting genetic data, in line with studies suggesting that splicing defects might be more widespread than initially presumed. To this regard, it is interesting to note the *PUS3* variant, c.497G > A, which was initially reported in several patients worldwide as a missense p.(Arg166Gln) change (Aldinger et al. 2019; de Paiva et al. 2019). Our functional analysis, however, showed that this variant activates a cryptic donor splice site, which ultimately causes an out-of-frame exon 3 deletion. This finding suggests a critical re-interpretation of the variant's effect from a simple amino acid change to a more severe splicing defect, illustrating the value of functional validation in refining genotype–phenotype correlations (Lin et al. 2022).

Our reassessment of *MID2* data reported in the literature strongly suggests that the initial evidence of its role in NDD is no longer supported by data: (i) the variant initially reported associated with NDD is present among controls (gnomAD v.4.1); (ii) *MID2* is not depleted for LoF variants (pLI = 0.18 gnomAD v.4.1), suggesting that haploinsufficiency is an unlikely pathogenic mechanism (Fig. S7b); (iii) a second *MID2* variant (NM_012216.4:c.166C > T p.(Arg56Cys) has been described more recently in one of two siblings, whose condition was associated with a homozygous pathogenic variant in *TRAPPC9*. The *MID2* variant was suspected to exacerbate the phenotype of the male proband (Kharrat et al. 2024), but this finding is questionable. Taken together, the current evidence for the pathogenicity of *MID2* variants is limited, and definitive proof of the involvement of this gene in NDDs remains lacking.

The identification of Jansen-de Vries syndrome cases led us to further investigate the role of LoF variants within *PPM1D.* The interpretation of truncating variants in this gene presents a significant challenge for neurodevelopmental diagnostics due to the phenomenon of Clonal Hematopoiesis of Indeterminate Potential (CHIP). While germline *PPM1D* mutations are established causes of syndromic NDD, identical truncating variants are frequently detected at low variant allele fractions (VAF) in the blood of healthy, typically older individuals (Zhang et al. 2022). These somatic mosaic events are primarily localized within the terminal exons of *PPM1D*. Because they escape nonsense-mediated decay (NMD), they result in a stable, truncated protein with a gain-of-function effect that confers a selective proliferative advantage to hematopoietic stem cells. This somatic 'contamination' in population databases can skew constraint metrics and complicate variant prioritization. Similar patterns of age-related somatic mosaicism affecting NDD-associated genes have been well-documented in other epigenetic and signaling regulators, most notably *ASXL1, DNMT3A, TET2,* and *GNB1* (Gudmundsson et al. 2022).

Another example of a potential novel gene-disease association involves *DSCAM*. We identified a patient with a *de novo* stop-gain variant in this gene. We noted that, despite a SFARI 1 (high confidence) classification, *DSCAM* is not listed as being associated with a disease in OMIM, and a clear link to NDD has not been established. *DSCAM* plays a role in neural development, including axon guidance, synaptic formation, and circuit assembly. The gene is located on chromosome 21, and overexpression has originally been implicated in Down syndrome. Studies have suggested that increased dosage and overexpression of the *DSCAM* gene may contribute to abnormal brain development, potentially linking it to ASD (Chen et al. 2022). Disruptions in these processes are believed to contribute to the etiology of ASD, providing a functional rationale for studying *DSCAM* in this context. Research involving animal models, particularly mice, has demonstrated that alterations in *DSCAM* expression can impact social behavior and brain connectivity. These behavioral changes in animal models offer parallels to human ASD symptoms, suggesting a possible connection. Furthermore, several studies have cited literature cases with LoF variants supporting its role in ASD (Iossifov et al. 2014; Lim et al. 2021; Monies et al. 2017; Wang et al. 2016), and pLI for the gene is 1 (gnomAD v.4.1). These findings underscore the importance of further research on *DSCAM* to clarify its potential link to ASD.

In conclusion, this research underscores the remarkable complexity of genetic underpinnings in NDDs, demonstrating varied inheritance patterns, identifying novel candidate genes, and revising standing gene-disease associations. Our findings solidify the role of ES as a front-line diagnostic tool in NDDs while calling for cautious interpretation of genetic data considering multifactorial phenotypic expressions. We recognize that the integration of novel "omics" technologies—including whole-genome sequencing (WGS), long-read sequencing (LRS), and transcriptomics—is becoming essential to resolve the most challenging cases. Recent studies have demonstrated that LRS, in particular, can significantly improve the detection of complex SVs and tandem repeat expansions that are refractory to short-read technologies (Steyaert et al. 2025). Transitioning toward these comprehensive genomic approaches will be a critical step in further reducing the diagnostic odyssey for the remaining 63.5% of our unsolved cohort. Future studies focusing on extended family analyses, larger cohorts, and functional assays will be crucial in further deciphering the intricate genetic framework guiding neurodevelopmental disorders, ultimately benefiting clinical diagnostic approaches. The integrated strategy of manually reviewing each variant in conjunction with deep phenotyping demonstrates its significant power as a diagnostic tool, which is the basis of precision medicine.

## Supplementary Information

Below is the link to the electronic supplementary material.

Supplementary Material 1

Supplementary Material 2

## Data Availability

The data analysed during this study can be found within the published article, its supplementary files, or available from authors upon reasonable request.

## References

[CR1] Aldinger KA, Timms AE, Thomson Z, Mirzaa GM, Bennett JT, Rosenberg AB, Roco CM, Hirano M, Abidi F, Haldipur P, Cheng CV, Collins S, Park K, Zeiger J, Overmann LM, Alkuraya FS, Biesecker LG, Braddock SR, Cathey S, Cho MT, Chung BHY, Everman DB, Zarate YA, Jones JR, Schwartz CE, Goldstein A, Hopkin RJ, Krantz ID, Ladda RL, Leppig KA, McGillivray BC, Sell S, Wusik K, Gleeson JG, Nickerson DA, Bamshad MJ, Gerrelli D, Lisgo SN, Seelig G, Ishak GE, Barkovich AJ, Curry CJ, Glass IA, Millen KJ, Doherty D, Dobyns WB (2019) Redefining the etiologic landscape of cerebellar malformations. Am J Hum Genet 105:606–615. 10.1016/j.ajhg.2019.07.01931474318 10.1016/j.ajhg.2019.07.019PMC6731369

[CR2] Antaki D, Guevara J, Maihofer AX, Klein M, Gujral M, Grove J, Carey CE, Hong O, Arranz MJ, Hervas A, Corsello C, Vaux KK, Muotri AR, Iakoucheva LM, Courchesne E, Pierce K, Gleeson JG, Robinson EB, Nievergelt CM, Sebat J (2022) A phenotypic spectrum of autism is attributable to the combined effects of rare variants, polygenic risk and sex. Nat Genet 54:1284–1292. 10.1038/s41588-022-01064-535654974 10.1038/s41588-022-01064-5PMC9474668

[CR3] Bain JM, Cho MT, Telegrafi A, Wilson A, Brooks S, Botti C, Gowans G, Autullo LA, Krishnamurthy V, Willing MC, Toler TL, Ben-Zev B, Elpeleg O, Shen Y, Retterer K, Monaghan KG, Chung WK (2016) Variants in HNRNPH2 on the X chromosome are associated with a neurodevelopmental disorder in females. Am J Hum Genet 99:728–734. 10.1016/j.ajhg.2016.06.02827545675 10.1016/j.ajhg.2016.06.028PMC5011042

[CR4] Bakur K, Hamid H, Alhaddad B, Alfadhel M, Alhashem A, Eyaid W, Alanzi T, Al Mutairi F, Alswaid A, Ababneh F, Al Ghamdi M, Mohamed S, Alaskar A, Alqahtani F, Alzaidan H, Al-Owain M, Faqeih EA, Mushiba AM, Alanazi R, Almoallem B, Alsaleh NS, Al Tala S, Alshammari M, Turkistani A, Gosadi G, Hakami F, Alobaid F, Al Rukban H, Alfaidi A, Ba-Abbad R, Almuqbil MA, Al-Boukai A, Alamri AS, Alshehri A, Sulaiman RA, Almontasheri A, Danish E, AlSagheir A, Aljeaid D, Al-Awam BS, Shawli A, Al-Otaibi M, Majdali WS, Azher ZA, Almannai M, Baalawi W, AlAbdi L, Benoukraf T, Alkuraya FS, Group SAG (2025) Adult genomic medicine: lessons from a multisite study of 2700 patients. Genome Med 17:105. 10.1186/s13073-025-01529-241024252 10.1186/s13073-025-01529-2PMC12477811

[CR5] Bauer CK, Calligari P, Radio FC, Caputo V, Dentici ML, Falah N, High F, Pantaleoni F, Barresi S, Ciolfi A, Pizzi S, Bruselles A, Person R, Richards S, Cho MT, Claps Sepulveda DJ, Pro S, Battini R, Zampino G, Digilio MC, Bocchinfuso G, Dallapiccola B, Stella L, Tartaglia M (2018) Mutations in KCNK4 that affect gating cause a recognizable neurodevelopmental syndrome. Am J Hum Genet 103:621–630. 10.1016/j.ajhg.2018.09.00130290154 10.1016/j.ajhg.2018.09.001PMC6174320

[CR6] Bosch E, Güse E, Kirchner P, Winterpacht A, Walther M, Alders M, Kerkhof J, Ekici AB, Sticht H, Sadikovic B, Reis A, Vasileiou G (2024) The missing link: ARID1B non-truncating variants causing Coffin-Siris syndrome due to protein aggregation. Hum Genet 143:965–978. 10.1007/s00439-024-02688-939028335 10.1007/s00439-024-02688-9PMC11303441

[CR7] Chen P, Liu Z, Zhang Q, Lin D, Song L, Liu J, Jiao HF, Lai X, Zou S, Wang S, Zhou T, Li BM, Zhu L, Pan BX, Fei E (2022) DSCAM deficiency leads to premature spine maturation and autism-like behaviors. J Neurosci 42:532–551. 10.1523/JNEUROSCI.1003-21.202134848499 10.1523/JNEUROSCI.1003-21.2021PMC8805618

[CR8] Chen S, Francioli LC, Goodrich JK, Collins RL, Kanai M, Wang Q, Alföldi J, Watts NA, Vittal C, Gauthier LD, Poterba T, Wilson MW, Tarasova Y, Phu W, Grant R, Yohannes MT, Koenig Z, Farjoun Y, Banks E, Donnelly S, Gabriel S, Gupta N, Ferriera S, Tolonen C, Novod S, Bergelson L, Roazen D, Ruano-Rubio V, Covarrubias M, Llanwarne C, Petrillo N, Wade G, Jeandet T, Munshi R, Tibbetts K, O’Donnell-Luria A, Solomonson M, Seed C, Martin AR, Talkowski ME, Rehm HL, Daly MJ, Tiao G, Neale BM, MacArthur DG, Karczewski KJ, Consortium GAD (2024) A genomic mutational constraint map using variation in 76,156 human genomes. Nature 625:92–100. 10.1038/s41586-023-06045-038057664 10.1038/s41586-023-06045-0PMC11629659

[CR9] Cingolani P, Platts A, Wang leL, Coon M, Nguyen T, Wang L, Land SJ, Lu X, Ruden DM (2012) A program for annotating and predicting the effects of single nucleotide polymorphisms, SnpEff: SNPs in the genome of *Drosophila melanogaster* strain w1118; iso-2; iso-3. Fly 6:80–92. 10.4161/fly.19695. (**19695 [pii]**)22728672 10.4161/fly.19695PMC3679285

[CR10] Cirnigliaro M, Chang TS, Arteaga SA, Pérez-Cano L, Ruzzo EK, Gordon A, Bicks LK, Jung JY, Lowe JK, Wall DP, Geschwind DH (2023) The contributions of rare inherited and polygenic risk to ASD in multiplex families. Proc Natl Acad Sci U S A 120:e2215632120. 10.1073/pnas.221563212037506195 10.1073/pnas.2215632120PMC10400943

[CR11] Cunningham JL, Frankovich J, Dubin RA, Pedrosa E, Baykara RN, Schlenk NC, Maqbool SB, Dolstra H, Marino J, Edinger J, Shea JM, Laje G, Swagemakers SMA, Sinnadurai S, Zhang ZD, Lin JR, van der Spek PJ, Lachman HM (2024) Ultrarare variants in DNA damage repair genes in pediatric acute-onset neuropsychiatric syndrome or acute behavioral regression in neurodevelopmental disorders. Dev Neurosci. 10.1159/00054190839396515 10.1159/000541908PMC12360735

[CR12] de Paiva ARB, Lynch DS, Melo US, Lucato LT, Freua F, de Assis BDR, Barcelos I, Listik C, de Castro Dos Santos D, Macedo-Souza LI, Houlden H, Kok F (2019) Mutations are associated with intellectual disability, leukoencephalopathy, and nephropathy. Neurol Genet 5:e306. 10.1212/NXG.000000000000030630697592 10.1212/NXG.0000000000000306PMC6340380

[CR13] De Rubeis S, He X, Goldberg AP, Poultney CS, Samocha K, Cicek AE, Kou Y, Liu L, Fromer M, Walker S, Singh T, Klei L, Kosmicki J, Shih-Chen F, Aleksic B, Biscaldi M, Bolton PF, Brownfeld JM, Cai J, Campbell NG, Carracedo A, Chahrour MH, Chiocchetti AG, Coon H, Crawford EL, Curran SR, Dawson G, Duketis E, Fernandez BA, Gallagher L, Geller E, Guter SJ, Hill RS, Ionita-Laza J, Jimenz Gonzalez P, Kilpinen H, Klauck SM, Kolevzon A, Lee I, Lei I, Lei J, Lehtimaki T, Lin CF, Ma’ayan A, Marshall CR, McInnes AL, Neale B, Owen MJ, Ozaki N, Parellada M, Parr JR, Purcell S, Puura K, Rajagopalan D, Rehnstrom K, Reichenberg A, Sabo A, Sachse M, Sanders SJ, Schafer C, Schulte-Ruther M, Skuse D, Stevens C, Szatmari P, Tammimies K, Valladares O, Voran A, Li-San W, Weiss LA, Willsey AJ, Yu TW, Yuen RK, Study DDD, Homozygosity Mapping Collaborative for A, Consortium UK, Cook EH, Freitag CM, Gill M, Hultman CM, Lehner T, Palotie A, Schellenberg GD, Sklar P, State MW, Sutcliffe JS, Walsh CA, Scherer SW, Zwick ME, Barett JC, Cutler DJ, Roeder K, Devlin B, Daly MJ, Buxbaum JD (2014) Synaptic, transcriptional and chromatin genes disrupted in autism. Nature 515:209–215. 10.1038/nature1377225363760 10.1038/nature13772PMC4402723

[CR14] Dougherty JD, Marrus N, Maloney SE, Yip B, Sandin S, Turner TN, Selmanovic D, Kroll KL, Gutmann DH, Constantino JN, Weiss LA (2022) Can the “female protective effect” liability threshold model explain sex differences in autism spectrum disorder? Neuron 110:3243–3262. 10.1016/j.neuron.2022.06.02035868305 10.1016/j.neuron.2022.06.020PMC9588569

[CR15] Fu JM, Satterstrom FK, Peng M, Brand H, Collins RL, Dong S, Wamsley B, Klei L, Wang L, Hao SP, Stevens CR, Cusick C, Babadi M, Banks E, Collins B, Dodge S, Gabriel SB, Gauthier L, Lee SK, Liang L, Ljungdahl A, Mahjani B, Sloofman L, Smirnov AN, Barbosa M, Betancur C, Brusco A, Chung BHY, Cook EH, Cuccaro ML, Domenici E, Ferrero GB, Gargus JJ, Herman GE, Hertz-Picciotto I, Maciel P, Manoach DS, Passos-Bueno MR, Persico AM, Renieri A, Sutcliffe JS, Tassone F, Trabetti E, Campos G, Cardaropoli S, Carli D, Chan MCY, Fallerini C, Giorgio E, Girardi AC, Hansen-Kiss E, Lee SL, Lintas C, Ludena Y, Nguyen R, Pavinato L, Pericak-Vance M, Pessah IN, Schmidt RJ, Smith M, Costa CIS, Trajkova S, Wang JYT, Yu MHC, Cutler DJ, De Rubeis S, Buxbaum JD, Daly MJ, Devlin B, Roeder K, Sanders SJ, Talkowski ME, (ASC) ASC, (Broad-CCDG) BICfCDG, Consortium i-B (2022) Rare coding variation provides insight into the genetic architecture and phenotypic context of autism. Nat Genet 54:1320–1331. 10.1038/s41588-022-01104-035982160 10.1038/s41588-022-01104-0PMC9653013

[CR16] Geetha TS, Michealraj KA, Kabra M, Kaur G, Juyal RC, Thelma BK (2014) Targeted deep resequencing identifies MID2 mutation for X-linked intellectual disability with varied disease severity in a large kindred from India. Hum Mutat 35:41–44. 10.1002/humu.2245324115387 10.1002/humu.22453

[CR17] Gilissen C, Hehir-Kwa JY, Thung DT, van de Vorst M, van Bon BW, Willemsen MH, Kwint M, Janssen IM, Hoischen A, Schenck A, Leach R, Klein R, Tearle R, Bo T, Pfundt R, Yntema HG, de Vries BB, Kleefstra T, Brunner HG, Vissers LE, Veltman JA (2014) Genome sequencing identifies major causes of severe intellectual disability. Nature 511:344–347. 10.1038/nature13394. (**nature13394 [pii]**)24896178 10.1038/nature13394

[CR18] Gudmundsson S, Singer-Berk M, Watts NA, Phu W, Goodrich JK, Solomonson M, Rehm HL, MacArthur DG, O’Donnell-Luria A, Consortium GAD (2022) Variant interpretation using population databases: lessons from gnomAD. Hum Mutat 43:1012–1030. 10.1002/humu.2430934859531 10.1002/humu.24309PMC9160216

[CR19] Ham H, Jing H, Lamborn IT, Kober MM, Koval A, Berchiche YA, Anderson DE, Druey KM, Mandl JN, Isidor B, Ferreira CR, Freeman AF, Ganesan S, Karsak M, Mustillo PJ, Teo J, Zolkipli-Cunningham Z, Chatron N, Lecoquierre F, Oler AJ, Schmid JP, Kuhns DB, Xu X, Hauck F, Al-Herz W, Wagner M, Terhal PA, Muurinen M, Barlogis V, Cruz P, Danielson J, Stewart H, Loid P, Rading S, Keren B, Pfundt R, Zarember KA, Vill K, Potocki L, Olivier KN, Lesca G, Faivre L, Wong M, Puel A, Chou J, Tusseau M, Moutsopoulos NM, Matthews HF, Simons C, Taft RJ, Soldatos A, Masle-Farquhar E, Pittaluga S, Brink R, Fink DL, Kong HH, Kabat J, Kim WS, Bierhals T, Meguro K, Hsu AP, Gu J, Stoddard J, Banos-Pinero B, Slack M, Trivellin G, Mazel B, Soomann M, Li S, Watts VJ, Stratakis CA, Rodriguez-Quevedo MF, Bruel AL, Lipsanen-Nyman M, Saultier P, Jain R, Lehalle D, Torres D, Sullivan KE, Barbarot S, Neu A, Duffourd Y, Similuk M, McWalter K, Blanc P, Bézieau S, Jin T, Geha RS, Casanova JL, Makitie OM, Kubisch C, Edery P, Christodoulou J, Germain RN, Goodnow CC, Sakmar TP, Billadeau DD, Küry S, Katanaev VL, Zhang Y et al (2024) Germline mutations in a G protein identify signaling cross-talk in T cells. Science 385:eadd8947. 10.1126/science.add894739298586 10.1126/science.add8947PMC11811912

[CR20] Hamada N, Negishi Y, Mizuno M, Miya F, Hattori A, Okamoto N, Kato M, Tsunoda T, Yamasaki M, Kanemura Y, Kosaki K, Tabata H, Saitoh S, Nagata KI (2017) Role of a heterotrimeric G-protein, Gi2, in the corticogenesis: possible involvement in periventricular nodular heterotopia and intellectual disability. J Neurochem 140:82–95. 10.1111/jnc.1387827787898 10.1111/jnc.13878

[CR21] Harmsen S, Buchert R, Mayatepek E, Haack TB, Distelmaier F (2019) Bain type of X-linked syndromic mental retardation in boys. Clin Genet 95:734–735. 10.1111/cge.1352430887513 10.1111/cge.13524

[CR22] Hiatt SM, Trajkova S, Sebastiano MR, Partridge EC, Abidi FE, Anderson A, Ansar M, Antonarakis SE, Azadi A, Bachmann-Gagescu R, Bartuli A, Benech C, Berkowitz JL, Betti MJ, Brusco A, Cannon A, Caron G, Chen Y, Cochran ME, Coleman TF, Crenshaw MM, Cuisset L, Curry CJ, Darvish H, Demirdas S, Descartes M, Douglas J, Dyment DA, Elloumi HZ, Ermondi G, Faoucher M, Farrow EG, Felker SA, Fisher H, Hurst ACE, Joset P, Kelly MA, Kmoch S, Leadem BR, Lyons MJ, Macchiaiolo M, Magner M, Mandrile G, Mattioli F, McEown M, Meadows SK, Medne L, Meeks NJL, Montgomery S, Napier MP, Natowicz M, Newberry KM, Niceta M, Noskova L, Nowak CB, Noyes AG, Osmond M, Prijoles EJ, Pugh J, Pullano V, Quélin C, Rahimi-Aliabadi S, Rauch A, Redon S, Reymond A, Schwager CR, Sellars EA, Scheuerle AE, Shukarova-Angelovska S, Skraban C, Stolerman E, Sullivan BR, Tartaglia M, Thiffault I, Uguen K, Umaña LA, van Bever Y, van der Crabben SN, van Slegtenhorst MA, Waisfisz Q, Washington C, Rodan LH, Myers RM, Cooper GM (2023) Deleterious, protein-altering variants in the transcriptional coregulator ZMYM3 in 27 individuals with a neurodevelopmental delay phenotype. Am J Hum Genet 110:215–227. 10.1016/j.ajhg.2022.12.00736586412 10.1016/j.ajhg.2022.12.007PMC9943726

[CR23] Iossifov I, O’Roak BJ, Sanders SJ, Ronemus M, Krumm N, Levy D, Stessman HA, Witherspoon KT, Vives L, Patterson KE, Smith JD, Paeper B, Nickerson DA, Dea J, Dong S, Gonzalez LE, Mandell JD, Mane SM, Murtha MT, Sullivan CA, Walker MF, Waqar Z, Wei L, Willsey AJ, Yamrom B, Lee YH, Grabowska E, Dalkic E, Wang Z, Marks S, Andrews P, Leotta A, Kendall J, Hakker I, Rosenbaum J, Ma B, Rodgers L, Troge J, Narzisi G, Yoon S, Schatz MC, Ye K, McCombie WR, Shendure J, Eichler EE, State MW, Wigler M (2014) The contribution of de novo coding mutations to autism spectrum disorder. Nature 515:216–221. 10.1038/nature1390825363768 10.1038/nature13908PMC4313871

[CR24] Jacquemont S, Coe BP, Hersch M, Duyzend MH, Krumm N, Bergmann S, Beckmann JS, Rosenfeld JA, Eichler EE (2014) A higher mutational burden in females supports a “female protective model” in neurodevelopmental disorders. Am J Hum Genet 94:415–425. 10.1016/j.ajhg.2014.02.00124581740 10.1016/j.ajhg.2014.02.001PMC3951938

[CR69] Jagadeesh KA, Wenger AM, Berger MJ, Guturu H, Stenson PD, Cooper DN, Bernstein JA, Bejerano G (2016) M-CAP eliminates a majority of variants of uncertain significance in clinical exomes at high sensitivity. Nat Genet 48(12):1581–1586. Epub 2016 Oct 24. PMID: 27776117. 10.1038/ng.370327776117 10.1038/ng.3703

[CR25] Jansen S, Geuer S, Pfundt R, Brough R, Ghongane P, Herkert JC, Marco EJ, Willemsen MH, Kleefstra T, Hannibal M, Shieh JT, Lynch SA, Flinter F, FitzPatrick DR, Gardham A, Bernhard B, Ragge N, Newbury-Ecob R, Bernier R, Kvarnung M, Magnusson EA, Wessels MW, van Slegtenhorst MA, Monaghan KG, de Vries P, Veltman JA, Lord CJ, Vissers LE, de Vries BB, Study DDD (2017) De novo truncating mutations in the last and penultimate exons of PPM1D cause an intellectual disability syndrome. Am J Hum Genet 100:650–658. 10.1016/j.ajhg.2017.02.00528343630 10.1016/j.ajhg.2017.02.005PMC5384016

[CR26] Johnston JJ, Teer JK, Cherukuri PF, Hansen NF, Loftus SK, Chong K, Mullikin JC, Biesecker LG, (NISC) NISC (2010) Massively parallel sequencing of exons on the X chromosome identifies RBM10 as the gene that causes a syndromic form of cleft palate. Am J Hum Genet 86:743–748. 10.1016/j.ajhg.2010.04.00720451169 10.1016/j.ajhg.2010.04.007PMC2868995

[CR27] Kharrat M, Triki C, Ben Isaa A, Bouchaala W, Alila O, Chouchen J, Ghouliya Y, Kamoun F, Tlili A, Fakhfakh F (2024) Expanding the genetic and phenotypic spectrum of TRAPPC9 and MID2-related neurodevelopmental disabilities: report of two novel mutations, 3D-modelling, and molecular docking studies. J Hum Genet 69:291–299. 10.1038/s10038-024-01242-938467738 10.1038/s10038-024-01242-9

[CR28] Khayat MM, Hu J, Jiang Y, Li H, Chander V, Dawood M, Hansen AW, Li S, Friedman J, Cross L, Bijlsma EK, Ruivenkamp CAL, Sansbury FH, Innis JW, O'Shea JO, Meng Q, Rosenfeld JA, McWalter K, Wangler MF, Lupski JR, Posey JE, Murdock D, Gibbs RA (2021) Missense mutations in Xia-Gibbs syndrome. HGG Adv. 10.1016/j.xhgg.2021.100049

[CR68] Kircher M, Witten DM, Jain P, O'Roak BJ, Cooper GM, Shendure J (2014) A general framework for estimating the relative pathogenicity of human genetic variants. Nat Genet 46(3):310–315. Epub 2014 Feb 2. PMID: 24487276; PMCID: PMC3992975. 10.1038/ng.289224487276 10.1038/ng.2892PMC3992975

[CR29] Koko M, Satterstrom FK, Warrier V, Martin H, Consortium AS, consortium A (2025) Contribution of autosomal rare and de novo variants to sex differences in autism. Am J Hum Genet. 10.1016/j.ajhg.2025.01.01639954678 10.1016/j.ajhg.2025.01.016PMC11947420

[CR30] Landrum MJ, Chitipiralla S, Kaur K, Brown G, Chen C, Hart J, Hoffman D, Jang W, Liu C, Maddipatla Z, Maiti R, Mitchell J, Rezaie T, Riley G, Song G, Yang J, Ziyabari L, Russette A, Kattman BL (2025) ClinVar: updates to support classifications of both germline and somatic variants. Nucleic Acids Res 53:D1313–D1321. 10.1093/nar/gkae109039578691 10.1093/nar/gkae1090PMC11701624

[CR31] Lek M, Karczewski KJ, Minikel EV, Samocha KE, Banks E, Fennell T, O’Donnell-Luria AH, Ware JS, Hill AJ, Cummings BB, Tukiainen T, Birnbaum DP, Kosmicki JA, Duncan LE, Estrada K, Zhao F, Zou J, Pierce-Hoffman E, Berghout J, Cooper DN, Deflaux N, DePristo M, Do R, Flannick J, Fromer M, Gauthier L, Goldstein J, Gupta N, Howrigan D, Kiezun A, Kurki MI, Moonshine AL, Natarajan P, Orozco L, Peloso GM, Poplin R, Rivas MA, Ruano-Rubio V, Rose SA, Ruderfer DM, Shakir K, Stenson PD, Stevens C, Thomas BP, Tiao G, Tusie-Luna MT, Weisburd B, Won HH, Yu D, Altshuler DM, Ardissino D, Boehnke M, Danesh J, Donnelly S, Elosua R, Florez JC, Gabriel SB, Getz G, Glatt SJ, Hultman CM, Kathiresan C, Laakso M, McCarroll S, McCarthy MI, McGovern D, McPherson R, Neale BM, Palotie A, Purcell SM, Saleheen D, Scharf JM, Sklar P, Sullivan PF, Tuomilehto J, Tsuang MT, Watkins HC, Wilson JG, Daly MJ, MacArthur DG, Exome Aggregation C (2016) Analysis of protein-coding genetic variation in 60,706 humans. Nature 536:285–291. 10.1038/nature1905727535533 10.1038/nature19057PMC5018207

[CR33] Li H, Durbin R (2009) Fast and accurate short read alignment with Burrows-Wheeler transform. Bioinformatics 25:1754–1760. 10.1093/bioinformatics/btp32419451168 10.1093/bioinformatics/btp324PMC2705234

[CR34] Li Q, Wang K (2017) InterVar: clinical interpretation of genetic variants by the 2015 ACMG-AMP guidelines. Am J Hum Genet 100:267–280. 10.1016/j.ajhg.2017.01.00428132688 10.1016/j.ajhg.2017.01.004PMC5294755

[CR35] Li Z, Du C, Zhang C, Zhang M, Ying Y, Liang Y, Luo X (2020) Novel truncating variant of PPM1D penultimate exon in a Chinese patient with Jansen-de Vries syndrome. Mol Genet Genomic Med 8:e1120. 10.1002/mgg3.112031916397 10.1002/mgg3.1120PMC7057113

[CR32] Li C, Zhi D, Wang K, Liu X (2022) MetaRNN: differentiating rare pathogenic and rare benign missense SNVs and InDels using deep learning. Genome Med 14:115. 10.1186/s13073-022-01120-z36209109 10.1186/s13073-022-01120-zPMC9548151

[CR36] Lim CS, Kim MJ, Choi JE, Islam MA, Lee YK, Xiong Y, Shim KW, Yang JE, Lee RU, Lee J, Park P, Kwak JH, Seo H, Kim CH, Lee JH, Lee YS, Hwang SK, Lee K, Lee JA, Kaang BK (2021) Dysfunction of NMDA receptors in neuronal models of an autism spectrum disorder patient with a DSCAM mutation and in Dscam-knockout mice. Mol Psychiatry 26:7538–7549. 10.1038/s41380-021-01216-934253863 10.1038/s41380-021-01216-9PMC8873012

[CR37] Lin TY, Smigiel R, Kuzniewska B, Chmielewska JJ, Kosińska J, Biela M, Biela A, Kościelniak A, Dobosz D, Laczmanska I, Chramiec-Głąbik A, Jeżowski J, Nowak J, Gos M, Rzonca-Niewczas S, Dziembowska M, Ploski R, Glatt S (2022) Destabilization of mutated human PUS3 protein causes intellectual disability. Hum Mutat 43:2063–2078. 10.1002/humu.2447136125428 10.1002/humu.24471PMC10092196

[CR38] Liu X, Wu C, Li C, Boerwinkle E (2016) dbNSFP v3.0: a one-stop database of functional predictions and annotations for human nonsynonymous and splice-site SNVs. Hum Mutat 37:235–241. 10.1002/humu.2293226555599 10.1002/humu.22932PMC4752381

[CR39] Majethia P, Harish R, Narayanan DL, B L Y, Sharma S, Shukla A (2023) Further evidence of biallelic variants in KCNK18 as a cause of intellectual disability and epilepsy with febrile seizure plus. Clin Dysmorphol 32: 147–150. 10.1097/MCD.000000000000046310.1097/MCD.0000000000000463PMC1052384937195340

[CR70] Mancini C, Vaula G, Scalzitti L, Cavalieri S, Bertini E, Aiello C, Lucchini C, Gatti RA, Brussino A, Brusco A (2012) Megalencephalic leukoencephalopathy with subcortical cysts type 1 (MLC1) due to a homozygous deep intronic splicing mutation (c.895-226T>G) abrogated in vitro using an antisense morpholino oligonucleotide. Neurogenetics. ;13(3):205-14. Epub 2012 May 3. PMID: 22552818. 10.1007/s10048-012-0331-z10.1007/s10048-012-0331-z22552818

[CR40] Mangano GD, Fontana A, Salpietro V, Antona V, Mangano GR, Nardello R (2022) Recurrent missense variant in the nuclear export signal of FMR1 associated with FXS-like phenotype including intellectual disability, ASD, facial abnormalities. Eur J Med Genet 65:104441. 10.1016/j.ejmg.2022.10444135091116 10.1016/j.ejmg.2022.104441

[CR41] Monies D, Abouelhoda M, AlSayed M, Alhassnan Z, Alotaibi M, Kayyali H, Al-Owain M, Shah A, Rahbeeni Z, Al-Muhaizea MA, Alzaidan HI, Cupler E, Bohlega S, Faqeih E, Faden M, Alyounes B, Jaroudi D, Goljan E, Elbardisy H, Akilan A, Albar R, Aldhalaan H, Gulab S, Chedrawi C, Al Saud BK, Kurdi W, Makhseed N, Alqasim T, El Khashab HY, Al-Mousa H, Alhashem A, Kanaan I, Algoufi T, Alsaleem K, Basha TA, Al-Murshedi F, Khan S, Al-Kindy A, Alnemer M, Al-Hajjar S, Alyamani S, Aldhekri H, Al-Mehaidib A, Arnaout R, Dabbagh O, Shagrani M, Broering D, Tulbah M, Alqassmi A, Almugbel M, AlQuaiz M, Alsaman A, Al-Thihli K, Sulaiman RA, Al-Dekhail W, Alsaegh A, Bashiri FA, Qari A, Alhomadi S, Alkuraya H, Alsebayel M, Hamad MH, Szonyi L, Abaalkhail F, Al-Mayouf SM, Almojalli H, Alqadi KS, Elsiesy H, Shuaib TM, Seidahmed MZ, Abosoudah I, Akleh H, AlGhonaium G, Alkharfy TM, Al Mutairi F, Eyaid W, Alshanbary A, Sheikh FR, Alsohaibani FI, Alsonbul A, Al Tala S, Balkhy S, Bassiouni R, Alenizi AS, Hussein MH, Hassan S, Khalil M, Tabarki B, Alshahwan S, Oshi A, Sabr Y, Alsaadoun S, Salih MA, Mohamed S, Sultana H, Tamim A, El-Haj M, Alshahrani S, Bubshait DK, Alfadhel M et al (2017) The landscape of genetic diseases in Saudi Arabia based on the first 1000 diagnostic panels and exomes. Hum Genet 136:921–939. 10.1007/s00439-017-1821-828600779 10.1007/s00439-017-1821-8PMC5502059

[CR42] Nakhleh Francis Y, Hershkovitz T, Ekhilevitch N, Habib C, Ravid S, Tal G, Schertz M, Mory A, Zinger A, Baris Feldman H, Zaid R, Paperna T, Weiss K (2023) Publicly funded exome sequencing for outpatients with neurodevelopmental disorders demonstrates a high rate of unexpected findings impacting medical management. Genet Med Open 1:100828. 10.1016/j.gimo.2023.10082839669259 10.1016/j.gimo.2023.100828PMC11613680

[CR43] Niceta M, Barresi S, Pantaleoni F, Capolino R, Dentici ML, Ciolfi A, Pizzi S, Bartuli A, Dallapiccola B, Tartaglia M, Digilio MC (2019) TARP syndrome: long-term survival, anatomic patterns of congenital heart defects, differential diagnosis and pathogenetic considerations. Eur J Med Genet 62:103534. 10.1016/j.ejmg.2018.09.00130189253 10.1016/j.ejmg.2018.09.001

[CR44] O’Roak BJ, Vives L, Fu W, Egertson JD, Stanaway IB, Phelps IG, Carvill G, Kumar A, Lee C, Ankenman K, Munson J, Hiatt JB, Turner EH, Levy R, O’Day DR, Krumm N, Coe BP, Martin BK, Borenstein E, Nickerson DA, Mefford HC, Doherty D, Akey JM, Bernier R, Eichler EE, Shendure J (2012) Multiplex targeted sequencing identifies recurrently mutated genes in autism spectrum disorders. Science 338:1619–1622. 10.1126/science.122776423160955 10.1126/science.1227764PMC3528801

[CR45] Parenti I, Rabaneda LG, Schoen H, Novarino G (2020) Neurodevelopmental disorders: from genetics to functional pathways. Trends Neurosci 43:608–621. 10.1016/j.tins.2020.05.00432507511 10.1016/j.tins.2020.05.004

[CR49] Pavinato L, Trajkova S, Grosso E, Giorgio E, Bruselles A, Radio FC, Pippucci T, Dimartino P, Tartaglia M, Petlichkovski A, De Rubeis S, Buxbaum J, Ferrero GB, Keller R, Brusco A (2021b) Expanding the clinical phenotype of the ultra-rare Skraban-Deardorff syndrome: two novel individuals with WDR26 loss-of-function variants and a literature review. Am J Med Genet A 185:1712–1720. 10.1002/ajmg.a.6215733675273 10.1002/ajmg.a.62157

[CR47] Pavinato L, Nematian-Ardestani E, Zonta A, De Rubeis S, Buxbaum J, Mancini C, Bruselles A, Tartaglia M, Pessia M, Tucker SJ, D'Adamo MC, Brusco A (2021a) Biallelic variants associated with intellectual disability and neurodevelopmental disorders alter TRESK channel activity. Int J Mol Sci. 10.3390/ijms2211606410.3390/ijms22116064PMC820003034199759

[CR48] Pavinato L, Stanic J, Barzasi M, Gurgone A, Chiantia G, Cipriani V, Eberini I, Palazzolo L, Di Luca M, Costa A, Marcantoni A, Biamino E, Spada M, Hiatt SM, Kelley WV, Vestito L, Sisodiya SM, Efthymiou S, Chand P, Kaiyrzhanov R, Bruselles A, Cardaropoli S, Tartaglia M, De Rubeis S, Buxbaum JD, Smedley D, Ferrero GB, Giustetto M, Gardoni F, Brusco A, Consortium GER (2023) Missense variants in RPH3A cause defects in excitatory synaptic function and are associated with a clinically variable neurodevelopmental disorder. Genet Med 25:100922. 10.1016/j.gim.2023.10092237403762 10.1016/j.gim.2023.100922

[CR46] Pavinato L, Carestiato S, Trajkova S, Sorasio L, Mantovani G, De Sanctis L, Kerkhof J, McConkey H, Rzasa J, Todd E, Balzo M, Cardaropoli S, Bruselles A, De Rubeis S, Buxbaum JD, Tartaglia M, Sadikovic B, Ferrero GB, Brusco A (2025) Skipping of Exon 20 in EP300: a novel variant linked to Rubinstein-Taybi syndrome with atypical and severe clinical manifestations. Clin Genet 107:354–358. 10.1111/cge.1465439603792 10.1111/cge.14654PMC11790522

[CR50] Posey JE, Harel T, Liu P, Rosenfeld JA, James RA, Coban Akdemir ZH, Walkiewicz M, Bi W, Xiao R, Ding Y, Xia F, Beaudet AL, Muzny DM, Gibbs RA, Boerwinkle E, Eng CM, Sutton VR, Shaw CA, Plon SE, Yang Y, Lupski JR (2017) Resolution of disease phenotypes resulting from multilocus genomic variation. N Engl J Med 376:21–31. 10.1056/NEJMoa151676727959697 10.1056/NEJMoa1516767PMC5335876

[CR51] Rees E, Creeth HDJ, Hwu HG, Chen WJ, Tsuang M, Glatt SJ, Rey R, Kirov G, Walters JTR, Holmans P, Owen MJ, O’Donovan MC (2021) Schizophrenia, autism spectrum disorders and developmental disorders share specific disruptive coding mutations. Nat Commun 12:5353. 10.1038/s41467-021-25532-434504065 10.1038/s41467-021-25532-4PMC8429694

[CR52] Satterstrom FK, Kosmicki JA, Wang J, Breen MS, De Rubeis S, An JY, Peng M, Collins R, Grove J, Klei L, Stevens C, Reichert J, Mulhern MS, Artomov M, Gerges S, Sheppard B, Xu X, Bhaduri A, Norman U, Brand H, Schwartz G, Nguyen R, Guerrero EE, Dias C, Betancur C, Cook EH, Gallagher L, Gill M, Sutcliffe JS, Thurm A, Zwick ME, Børglum AD, State MW, Cicek AE, Talkowski ME, Cutler DJ, Devlin B, Sanders SJ, Roeder K, Daly MJ, Buxbaum JD, Consortium AS, Consortium i-B (2020) Large-scale exome sequencing study implicates both developmental and functional changes in the neurobiology of autism. Cell 180:568-584.e23. 10.1016/j.cell.2019.12.03631981491 10.1016/j.cell.2019.12.036PMC7250485

[CR53] Savatt JM, Myers SM (2021) Genetic testing in neurodevelopmental disorders. Front Pediatr 9:526779. 10.3389/fped.2021.52677933681094 10.3389/fped.2021.526779PMC7933797

[CR54] Schaaf CP, Gonzalez-Garay ML, Xia F, Potocki L, Gripp KW, Zhang B, Peters BA, McElwain MA, Drmanac R, Beaudet AL, Caskey CT, Yang Y (2013) Truncating mutations of MAGEL2 cause Prader-Willi phenotypes and autism. Nat Genet 45:1405–1408. 10.1038/ng.277624076603 10.1038/ng.2776PMC3819162

[CR55] Smith ED, Blanco K, Sajan SA, Hunter JM, Shinde DN, Wayburn B, Rossi M, Huang J, Stevens CA, Muss C, Alcaraz W, Hagman KDF, Tang S, Radtke K (2019) A retrospective review of multiple findings in diagnostic exome sequencing: half are distinct and half are overlapping diagnoses. Genet Med 21:2199–2207. 10.1038/s41436-019-0477-230894705 10.1038/s41436-019-0477-2PMC6774997

[CR56] Srivastava S, Love-Nichols JA, Dies KA, Ledbetter DH, Martin CL, Chung WK, Firth HV, Frazier T, Hansen RL, Prock L, Brunner H, Hoang N, Scherer SW, Sahin M, Miller DT, Work NESR, Group (2019) Meta-analysis and multidisciplinary consensus statement: exome sequencing is a first-tier clinical diagnostic test for individuals with neurodevelopmental disorders. Genet Med 21:2413–2421. 10.1038/s41436-019-0554-631182824 10.1038/s41436-019-0554-6PMC6831729

[CR57] Stawiński P, Płoski R (2024) Genebe.net: implementation and validation of an automatic ACMG variant pathogenicity criteria assignment. Clin Genet 106:119–126. 10.1111/cge.1451638440907 10.1111/cge.14516

[CR58] Steyaert W, Sagath L, Demidov G, Yépez VA, Esteve-Codina A, Gagneur J, Ellwanger K, Derks R, Weiss M, den Ouden A, van den Heuvel S, Swinkels H, Zomer N, Steehouwer M, O’Gorman L, Astuti G, Neveling K, Schüle R, Xu J, Synofzik M, Beijer D, Hengel H, Schöls L, Claeys KG, Baets J, Van de Vondel L, Ferlini A, Selvatici R, Morsy H, Saeed Abd Elmaksoud M, Straub V, Müller J, Pini V, Perry L, Sarkozy A, Zaharieva I, Muntoni F, Bugiardini E, Polavarapu K, Horvath R, Reid E, Lochmüller H, Spinazzi M, Savarese M, Matalonga L, Laurie S, Brunner HG, Graessner H, Beltran S, Ossowski S, Vissers LELM, Gilissen C, Hoischen A, Solve-RD DITF-ITHACA S-RD-E-N, Solve-RD DITF-RND, S.lve-RD DITF-EpiCARE, consortium S-R (2025) Unraveling undiagnosed rare disease cases by HiFi long-read genome sequencing. Genome Res 35:755–768. 10.1101/gr.279414.12440138663 10.1101/gr.279414.124PMC12047270

[CR59] Trajkova S, Kerkhof J, Rossi Sebastiano M, Pavinato L, Ferrero E, Giovenino C, Carli D, Di Gregorio E, Marinoni R, Mandrile G, Palermo F, Carestiato S, Cardaropoli S, Pullano V, Rinninella A, Giorgio E, Pippucci T, Dimartino P, Rzasa J, Rooney K, McConkey H, Petlichkovski A, Pasini B, Sukarova-Angelovska E, Campbell CM, Metcalfe K, Jenkinson S, Banka S, Mussa A, Ferrero GB, Sadikovic B, Brusco A (2024) DNA methylation analysis in patients with neurodevelopmental disorders improves variant interpretation and reveals complexity. HGG Adv 5:100309. 10.1016/j.xhgg.2024.10030938751117 10.1016/j.xhgg.2024.100309PMC11216013

[CR60] Tsai MM, Lee NC, Chien YH, Hwu WL, Tung YC (2022) Short stature leads to a diagnosis of Jansen-de Vries syndrome in two unrelated Taiwanese girls: a case report and literature review. J Formos Med Assoc 121:856–860. 10.1016/j.jfma.2021.12.02235016835 10.1016/j.jfma.2021.12.022

[CR61] Turner TN, Eichler EE (2019) The role of de novo noncoding regulatory mutations in neurodevelopmental disorders. Trends Neurosci 42:115–127. 10.1016/j.tins.2018.11.00230563709 10.1016/j.tins.2018.11.002PMC6382467

[CR62] Verdura E, Rodríguez-Palmero A, Vélez-Santamaria V, Planas-Serra L, de la Calle I, Raspall-Chaure M, Roubertie A, Benkirane M, Saettini F, Pavinato L, Mandrile G, O’Leary M, O’Heir E, Barredo E, Chacón A, Michaud V, Goizet C, Ruiz M, Schlüter A, Rouvet I, Sala-Coromina J, Fossati C, Iascone M, Canonico F, Marcé-Grau A, de Souza P, Adams DR, Casasnovas C, Rehm HL, Mefford HC, González Gutierrez-Solana L, Brusco A, Koenig M, Macaya A, Pujol A (2021) Biallelic PI4KA variants cause a novel neurodevelopmental syndrome with hypomyelinating leukodystrophy. Brain 144:2659–2669. 10.1093/brain/awab12434415322 10.1093/brain/awab124PMC8557332

[CR64] Wang T, Guo H, Xiong B, Stessman HA, Wu H, Coe BP, Turner TN, Liu Y, Zhao W, Hoekzema K, Vives L, Xia L, Tang M, Ou J, Chen B, Shen Y, Xun G, Long M, Lin J, Kronenberg ZN, Peng Y, Bai T, Li H, Ke X, Hu Z, Zhao J, Zou X, Xia K, Eichler EE (2016) De novo genic mutations among a Chinese autism spectrum disorder cohort. Nat Commun 7:13316. 10.1038/ncomms1331627824329 10.1038/ncomms13316PMC5105161

[CR63] Wang J, Al-Ouran R, Hu Y, Kim SY, Wan YW, Wangler MF, Yamamoto S, Chao HT, Comjean A, Mohr SE, Perrimon N, Liu Z, Bellen HJ, UDN (2017) MARRVEL: integration of human and model organism genetic resources to facilitate functional annotation of the human genome. Am J Hum Genet 100:843–853. 10.1016/j.ajhg.2017.04.01028502612 10.1016/j.ajhg.2017.04.010PMC5670038

[CR71] Wiel L, Baakman C, Gilissen D, Veltman JA, Vriend G, Gilissen C, MetaDome (2019) Pathogenicity analysis of genetic variants through aggregation of homologous human protein domains. Hum Mutat 40(8):1030–1038. Epub 2019 Jun 18. PMID: 31116477; PMCID: PMC6772141. 10.1002/humu.2379831116477 10.1002/humu.23798PMC6772141

[CR65] Wojcik MH, Srivastava S, Agrawal PB, Balci TB, Callewaert B, Calvo PL, Carli D, Caudle M, Colaiacovo S, Cross L, Demetriou K, Drazba K, Dutra-Clarke M, Edwards M, Genetti CA, Grange DK, Hickey SE, Isidor B, Küry S, Lachman HM, Lavillaureix A, Lyons MJ, Marcelis C, Marco EJ, Martinez-Agosto JA, Nowak C, Pizzol A, Planes M, Prijoles EJ, Riberi R, Rush ET, Russell BE, Sachdev R, Schmalz B, Shears D, Stevenson DA, Wilson K, Jansen S, de Vries BBA, Curry CJ (2023) Jansen-de Vries syndrome: expansion of the PPM1D clinical and phenotypic spectrum in 34 families. Am J Med Genet A 191:1900–1910. 10.1002/ajmg.a.6322637183572 10.1002/ajmg.a.63226PMC10330231

[CR66] Zeidler S, Severijnen LA, de Boer H, van der Toorn EC, Ruivenkamp CAL, Bijlsma EK, Willemsen R (2021) A missense variant in the nuclear export signal of the FMR1 gene causes intellectual disability. Gene 768:145298. 10.1016/j.gene.2020.14529833181255 10.1016/j.gene.2020.145298

[CR67] Zhang L, Hsu JI, Goodell MA (2022) PPM1D in solid and hematologic malignancies: friend and foe? Mol Cancer Res 20:1365–1378. 10.1158/1541-7786.MCR-21-101835657598 10.1158/1541-7786.MCR-21-1018PMC9437564

