# Supplementary material for: The NeuroWES project: lessons learned from comprehensive phenotyping and genetic analysis of neurodevelopmental disorders over a decade
Source: Hum Genet. 2026 Jul 8;145(1):60. doi: 10.1007/s00439-026-02843-4 (PMC13346230; doi:10.1007/s00439-026-02843-4)
Supplement: Supplementary file 2 — Supplementary Material 2 [file 439_2026_2843_MOESM2_ESM.docx]

**Supplemental file**

**
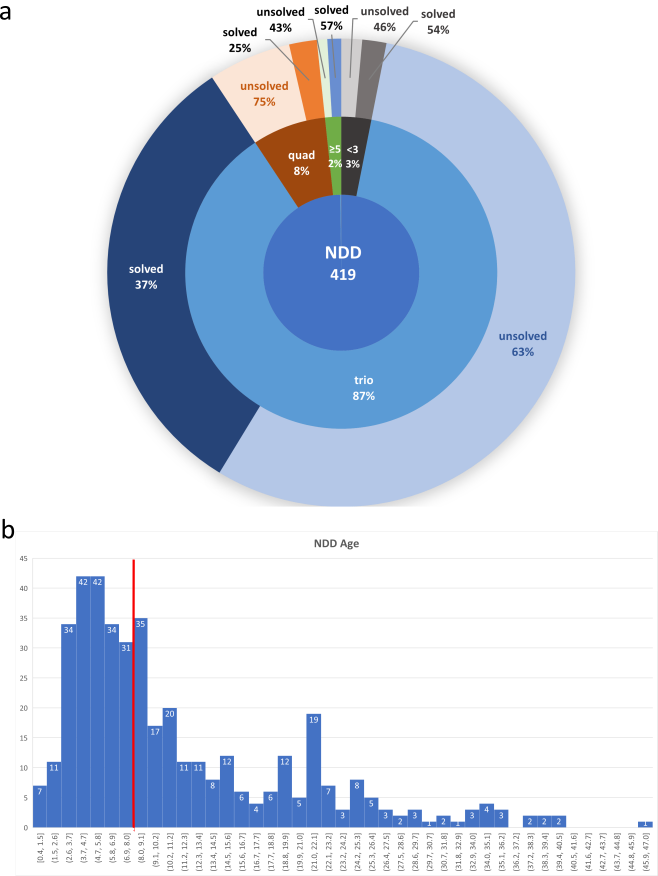
**

**Figure S1. Composition of family structures in the NeuroWES project**

Panel a. The pie-donut chart illustrates the distribution of family structures within the NeuroWES project. The second ring categorizes the families into trios, quads, or larger groups (≥5), including instances where one parent is missing (<3). The third ring indicates the percentage of cases identified as solved versus unsolved.

Panel b. The histogram displays the distribution of age for NDDs NeuroWES patients. The x-axis represents age bins, while the y-axis shows the count of individuals in each age category. The vertical red line indicates median (8 years). The highest counts are seen in younger age groups (early childhood), gradually decreasing as age increases.


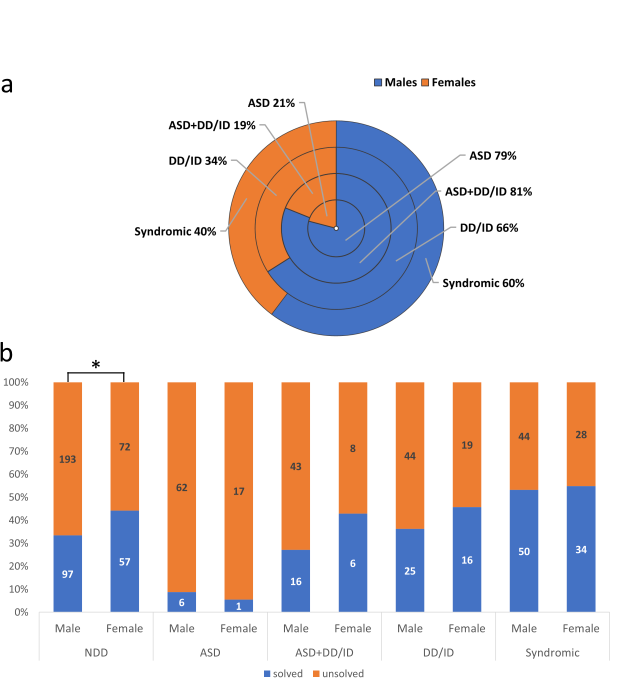


**Figure S2. Distribution of male vs. females in the NeuroWES database.**

Panel a. The pie-donut chart depicts the gender distribution within various subgroups of the NeuroWES database, highlighting differences between males and females. In cases of Autism Spectrum Disorder (ASD) and ASD combined with Developmental Delay or Intellectual Disability (ASD+DD/ID), the ratio of males to females was notably skewed, showing ratios of 1:4. In the subgroup of patients diagnosed solely with DD/ID the ratio was 1:2 and in Syndromic patients, the gender ratio approached a more balanced proportion of 1.5, with males slightly more represented.

Panel b. The stacked bar chart compares diagnostic data across the four subgroups of NeuroWES database—split between males and females. Each bar represents a 100% distribution of two statuses: "unsolved" (orange) and "solved" (blue). The ratio of "unsolved" to "solved" is higher in males than females across most categories. Across all patients in the NDD category, this difference was notably statistically significant (*p=0.039, χ2 test).

**
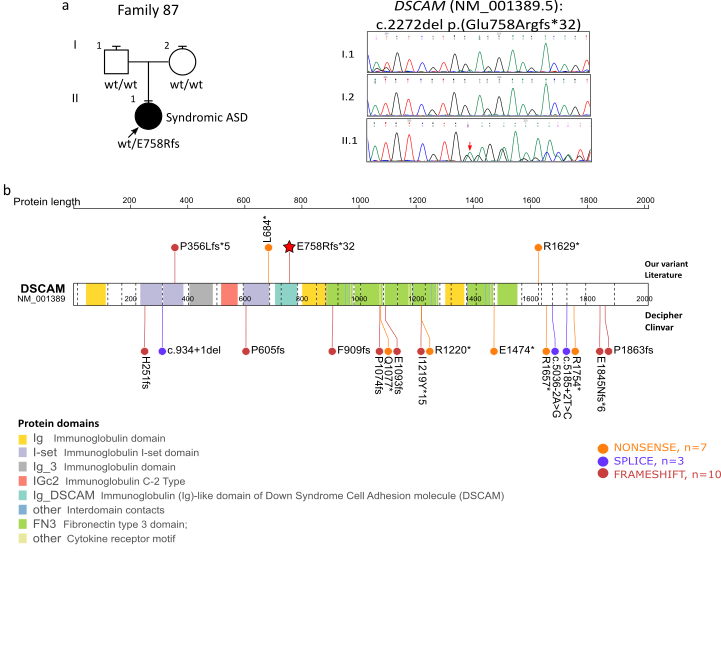
**

**Figure S3. *DSCAM* LoF variants.**

Panel a. The pedigree of family 87 illustrates that the NM_001389.5(*DSCAM*): c.2272del p.(Glu758Argfs*32) variant is *de novo*. In panels b, *DSCAM* variants reported in the literature (Guo et al. 2018; Lim et al. 2021; Wang et al. 2016), Decipher (504197, 510452, 519523, 531759, and ClinVar and our case variant are displayed alongside DSCAM protein (star symbol). Figure created with St. Jude Cloud PeCan (<https://pecan.stjude.cloud/variants/proteinpaint>). This gene is predicted to be constrained for LoF variants (pLI=1) and had been associated with ASD according to several studies (De Rubeis et al. 2014; Fu et al. 2022; Iossifov et al. 2014; Wang et al. 2016). An intronic SNP in this gene was linked to ASD in a GWAS meta-analysis of 7,387 ASD cases and 8,567 controls, with a *p*-value < 1.0x10^-4^  (Consortium 2017). Using Decipher, ClinVar, and published papers, we found 18 cases with monoallelic likely gene-disrupting variants (nonsense, frameshift, splicing in invariant sites). Clinical information about these cases is scanty, but available phenotypes almost always include ASD variably combined with ID.


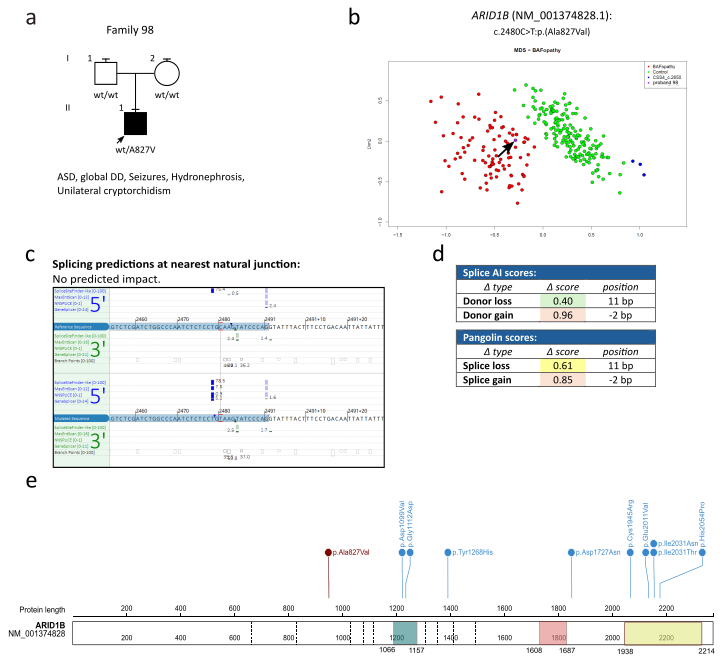


**Figure S4. *ARID1B* reported missense variants family 98**

Panel a. The pedigree of family 98 reveals the proband, who presents with ASD, DD, seizures, and genitourinary anomalies, carries a *de novo* *ARID1B* missense variant, p.(Ala827Val). *ARID1B* is associated with Coffin-Siris syndrome 1 (OMIM #135900), and pathogenic variants are predominantly loss-of-function (LoF), with rare missense changes reported mainly within the EHD1/2 and ARID domains (Bosch et al. 2024; Mermet-Meillon et al. 2024). Panel b. The methylation profile of this variant, p.(Ala827Val), notably matched the common BAF-opathy episignature, as previously published (Trajkova et al. 2024). MDS - multidimensional scaling plot. Green dots represent the control population; red dots represent patients with deleterious variants in genes coding for proteins in the BAFopathy complex; blue dots represent patients with the CSS4_c.2650 subsignature; the purple dot represents proband 98. Panel c and d. Bioinformatics predictions for the variant's impact on splicing were conflicting. Alamut 1.9 (<https://www.sophiagenetics.com/>) predicted no splicing impact (c), while SpliceAI (Jaganathan et al. 2019) and Pangolin (Zeng and Li 2022) predicted splice gain (d). Panel e, created with St. Jude Cloud PeCan (<https://pecan.stjude.cloud/variants/proteinpaint?gene=ARID1B>), illustrates the ARID1B protein with its domains: green box - ARID domain; red box - EHD1 domain; yellow box - EHD2 domain. Variant reported herein is shown in red, while variants reported in the literature are in blue. Protein domains were modified from (Bosch et al. 2024).


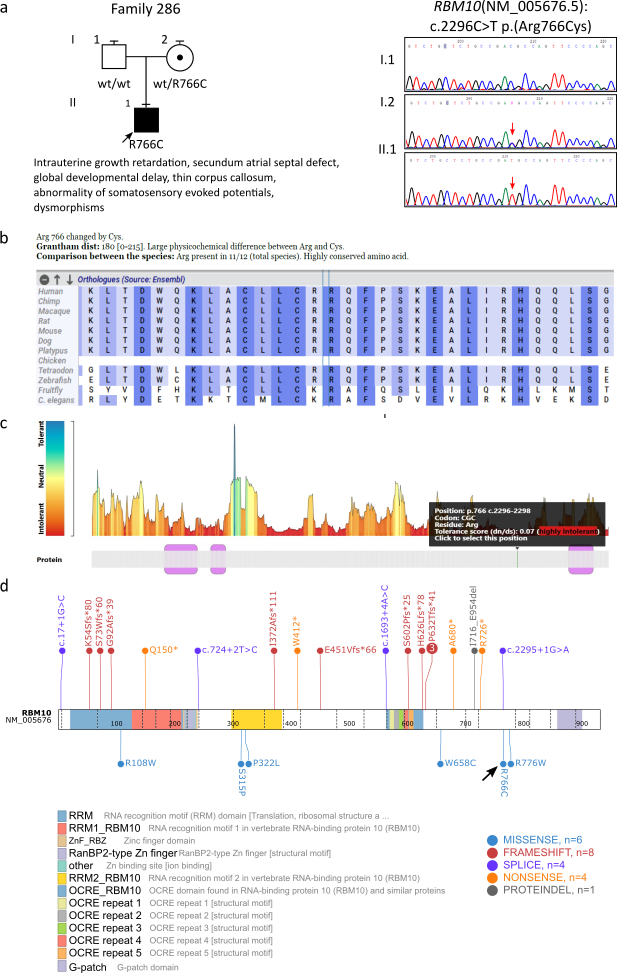


**Figure S5. *RBM10* family 286**

Panel a. The pedigree of family 286 demonstrates maternal inheritance of the NM_005676.5(*RBM10*): c.2296C>T variant.

Panel b. Cross-species conservation for the *RBM10* variant position is presented in Alamut 1.9 (<https://www.sophiagenetics.com/>).

Panel c. Analysis of the amino acid tolerance landscape of the *RBM10* protein using MetaDome (Wiel et al. 2019) predicts this specific position to be highly intolerant to variations. While *RBM10* is predominantly linked to NDDs through protein-truncating, loss-of-function variants (Højland et al. 2018; Johnston et al. 2010; Niceta et al. 2019), this missense variant, c.2296C>T [p.(Arg766Cys)], located within the C-terminal C2H2 Zinc Finger domain, shows compelling evidence of functional impact. Arginine 766 is a highly conserved residue (conserved in *C. elegans*). The substitution to Cysteine is supported by a high CADD (v1.6) Phred score of 31 and predicted high intolerance to variation by MetaDome. The variant's absence from gnomAD 4.1.0 and the gene's high missense Z score (4.46) further underscore the intolerance of *RBM10* to missense alterations.

Panel d, created with St. Jude Cloud PeCan (https://pecan.stjude.cloud/variants/proteinpaint?gene=rbm10), illustrates the RBM10 protein with its domains. The black arrow shows variant reported herein.


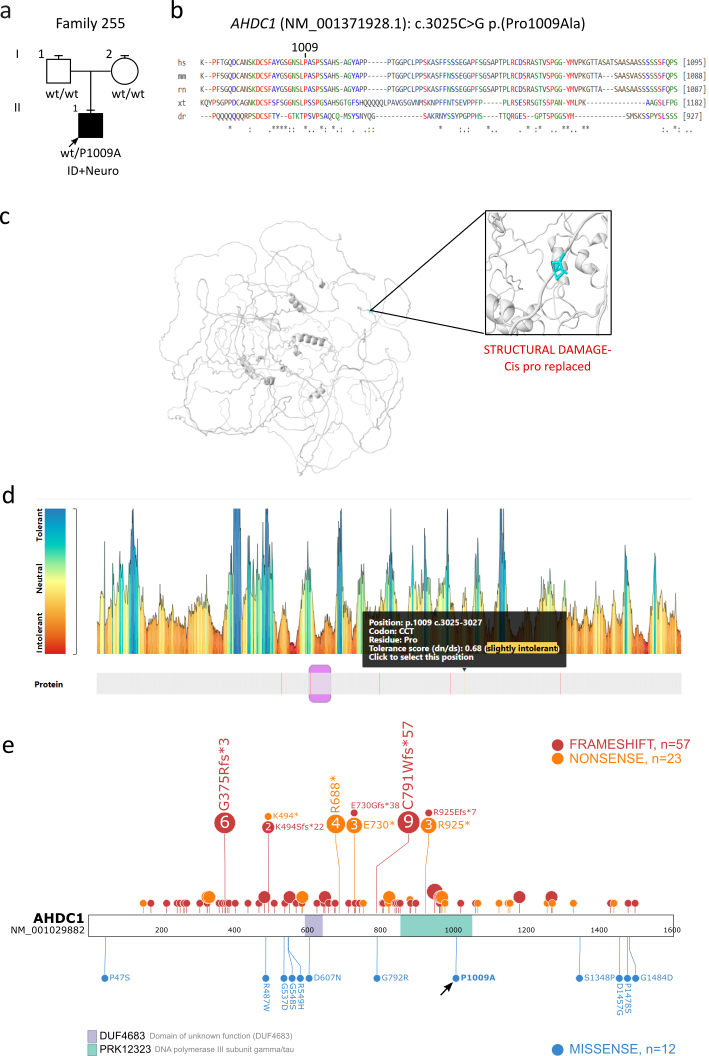


**Figure S6. *AHDC1* missense variant in family 255.**

Panel a. The pedigree of family 255 indicates the affected female proband carries a *de novo* variant in *AHDC1*, specifically NM_001371928.1: c.3025C>G, which results in the amino acid substitution p.(Pro1009Ala).

Panel b. Cross-species conservation for the *AHDC1* protein sequence (amino acids 985-1095) is shown using marrvel.org (<https://marrvel.org/>) (Wang et al. 2017).

Panel c. Modeling the p.(Pro1009Ala) variant with Missense3D (<https://missense3d.bc.ic.ac.uk/missense3d/>) (PDB: AF-Q5TGY3-F1-v4) predicted structural damage due to the replacement of a *cis*-proline.

Panel d. The MetaDome amino acid tolerance landscape for *AHDC1* (<https://stuart.radboudumc.nl/metadome/dashboard>) (Wiel et al. 2019) suggests this position has slight intolerance to variations. Xia-Gibbs syndrome is primarily associated with *de novo* truncating variants in *AHDC1* that lead to loss-of-function; however, missense variants have also been reported (Gumus 2020; Khayat et al. 2021). Proline 1009 is a highly conserved amino acid; MetaDome predicts slight intolerance to variation at this position. The clinical features observed in our patient, including DD/ID, hypotonia, seizures, sleep apnea, hypoplasia of the corpus callosum, atrial septal defect, and feeding difficulties, are consistent with the phenotype spectrum reported for individuals with *AHDC1* missense variants (Maleki et al. 2014).

Panel e, created with St. Jude Cloud PeCan (https://pecan.stjude.cloud/variants/proteinpaint?gene=AHDC1), illustrates the AHDC1 protein with its domains: violet box – DUF4683 domain; green box – PRK12323 domain. The black arrow shows variant reported herein.

**
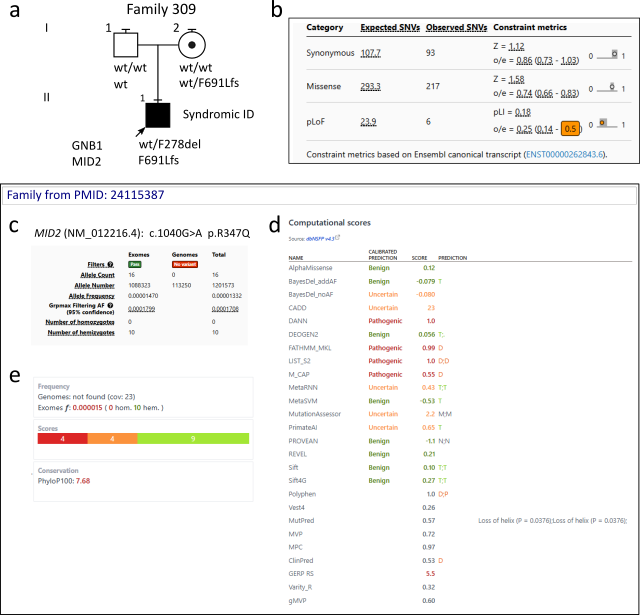
**

**Figure S7. Reevaluating the impact of *MID2* variants in NDDs.**

Panel a. The pedigree of family 309 demonstrates maternal inheritance of the NM_012216.4 (*MID2*): c.2070del variant. The proband also carries a pathogenic *GNB1* loss-of-function variant, which is consistent with the phenotype.

Panel b. GnomAD project (v4.1.0) constraint metrics predict *MID2* to be haplosufficient.

Panel c. *MID2* was associated to a neurodevelopmental disorders because of a c.1040G>A, p.(Arg347Gln) variant, initially reported in a large Indian family (Geetha et al. 2014). This variant is now reported in ten male subjects within gnomAD (v4.1.0).

In panels d and e, a summary available on the GeneBe website shows that most *in silico* tools predict a benign outcome for this variant.

| **Table S2. Primers for splicing effect validation at the RNA level.** | | |
| --- | --- | --- |
| Primers | Sequence | Variant |
| NFIB-ex1-F  NFIB-ex4-R | 5’-gaagattttcttgggcatctcc  5’-agttccctgggttatgggcg | *NFIB*(NM_001190737.2):  c.562G>C |
| PUS3-ex3F-EcoRI  PUS3-ex3R-BamHI | ggtGAATTCGTGCCTTTGGACAGGTAAGG  ggtGGATCCTGCAGTGGTCCAATCTCAGC | *PUS3*(NM_031307.4):  c.497G>A |
| SD6-F  SA2-R | 5’- TCTGAGTCACCTGGACAACC  5’- ATCTCAGTGGTATTTGTGAGC | pSPL3 vector |

| **Table S3. Recurrent variants** | | | | | | | |
| --- | --- | --- | --- | --- | --- | --- | --- |
| Family | Gene | NM_code | cDNA change | Protein change | Inheritance | ACMG classification | References  (PMID) |
| 407 | ***ACTB*** | NM_001101.5 | c.589G>A | p.(Gly197Ser) | *de novo* (AD) | LP | 25979418 |
| 251 | ***ANKRD11*** | NM_013275.6 | c.211_226+1del | p.? | *de novo* (AD) | P | 33476899 |
| 369 | ***CAMK2B*** | NM_001220.5 | c.416C>T | p.(Pro139Leu) | *de novo* (AD) | P | 32875707, 30842224, 29100089 |
| 425 | ***CHD7*** | NM_017780.4 | c.6194G>A | p.(Arg2065His) | *de novo* (AD) | LP | 25064402, 25383892, 30733481 |
| 298 | ***CNKSR2*** | NM_014927.5 | c.1198C>T | p.(Arg400*) | *de novo* (XLR) | P | 34266427 |
| 101 | ***CREBBP*** | NM_004380.3 | c.3779+1G>A | p.? | *de novo* (AD) | P | 27165009, 32827181 |
| 404 | ***DHDDS*** | NM_205861.3 | c.110G>A | p.(Arg37His) | *de novo* (AD) | P | 31780880, 29100083, 32654954 |
| 156 | ***DLG4*** | NM_001321075.3 | c.478G>T | p.(Glu160*) | *de novo* (AD) | P | 33597769 |
| 108 | ***EHMT1*** | NM_024757.5 | c.3000del | p.(Asp1001Thrfs*9) | *de novo* (AD) | P | 30525188 |
| 88 | ***FMR1*** | NM_002024.6 | c.1325G>A | p.(Arg442Gln) | maternal (XLD) | LP | 33181255 |
| 34 | ***FOXP1*** | NM_001349338.3 | c.1406C>T | p.(Thr469Ile) | *de novo* (AD) | LP | 31199603 |
| 202 | ***H3F3B*** | NM_005324.5 | c.377A>G | p.(Gln126Arg) | *de novo* (AD) | P | 33268356 |
| 186 | ***HNRNPH2*** | NM_019597.5 | c.617G>A | p.(Arg206Gln) | *de novo* (XLD) | LP | 27545675, 30887513 |
| 417 | ***KCNC2*** | NM_139137.4 | c.1408C>T | p.(Pro470Ser) | *de novo* (AD) | LP | 36087422 |
| 137 | ***KCNH1*** | NM_172362.3 | c.1486G>A | p.(Gly496Arg) | *de novo* (AD) | P | 33811134, 25915598 |
| 401 | ***KCNH5*** | NM_139318.5 | c.980G>A | p.(Arg327His) | *de novo* (AD) | P | 36307226, 35874597, 32725632 |
| 109 | ***MEIS2*** | NM_170675.5 | c.998_1000del | p.(Arg333del) | *de novo* (AD) | LP | 30291340, 30055086, 25712757 |
| 299 | ***NAA10*** | NM_003491.4 | c.247C>T | p.(Arg83Cys) | *de novo* (AD) | P | 37130971 |
| 302 | ***NAA15*** | NM_057175.5 | c.382C>T | p.(Arg128*) | *de novo* (AD) | P | 30792901 |
| 245  424 | ***PACS1*** | NM_018026.4 | c.607C>T | p.(Arg203Trp) | *de novo* (AD) | LP | 36645641 |
| 383 | ***PIK3CA*** | NM_006218.4 | c.278G>A | p.(Arg93Gln) | *de novo* (AD) | LP | 27631024 |
| 188 | ***PPM1D*** | NM_003620.4 | c.1280G>A | p.(Trp427*) | *de novo* (AD) | LP | 28343630 |
| 91 | ***PRR12*** | NM_020719.3 | c.3273del | p.(Lys1092Argfs*131) | *de novo* (AD) | P | 33824499 |
| 175 | ***PTPN11*** | NM_002834.5 | c.209A>G | p.(Lys70Arg) | *de novo* (AD) | P | 29084544, 34346503 |
| 213 | ***SHANK3*** | NM_001080420.1 | c.3727dup | p.(Ala1243Glyfs*69) | *de novo* (AD) | P | 30763456, 29719671, 25356970 |
| 7 | ***TLK2*** | NM_006852.6 | c.1586A>G | p.(Asp529Gly) | *de novo* (AD) | LP | 33323470 |
| 139 | ***WDR26*** | NM_001379403.1 | c.1376G>A | p.(Trp459*) | *de novo* (AD) | P | 33675273 |
| 103 | ***WDR37*** | NM_014023.4 | c.356C>T | p.(Ser119Phe) | *de novo* (AD) | LP | 31327508, 31327510 |

| **Table S4. Dual genetic diagnoses** | | | | | | | |
| --- | --- | --- | --- | --- | --- | --- | --- |
| **Family** | **Gene 1** | **Inheritance/ACMG classification 1** | ***Phenotype 1** | **Gene 2** | **Inheritance/ACMG classification 2** | ***Phenotype 2** | **Summary**  and  **Additional Phenotype** |
| 82 | ***CAPN10*** | homozygous (AR)/Pathogenic | Global Developmental Delay HP:0001263, Microcephaly HP:0000252, Absent speech HP:0001344 (Oladnabi et al. 2015) | ***RPL10*** | maternal (XLR)/VUS | Global Developmental Delay HP:0001263, Microcephaly HP:0000252, Absent speech HP:0001344, Motor delay HP:0001270 | Overlapping |
| 45 | ***FOXP1*** | *de novo* (AD)/P | Autistic behavior HP:0000729, Global Developmental Delay HP:0001263, Strabismus HP:0000486, Absent speech HP:0001344, Hyperactivity HP:0000752, Punctate periventricular T2 hyperintense foci HP:0030081, Abnormality of the face HP:0000271 | ***DLG3*** | maternal (XLR)/VUS | Autistic behavior HP:0000729, Global Developmental Delay HP:0001263, Strabismus HP:0000486, Absent speech HP:0001344, Hyperactivity HP:0000752, Abnormality of the face HP:0000271, Hypotonia HP:0001252 | Overlapping |
| 309 | ***GNB1*** | *de novo* (AD)/LP | Global Developmental Delay HP:0001263, Delayed fine motor development HP:0010862, Intellectual disability HP:0001249, Impaired social interactions HP:0000735, Behavioral abnormality HP:0000708, Bilateral conductive hearing impairment HP:0008513 (mild) (Nasvytis et al. 2024) | ***MID2*** | maternal (XLR)/VUS | Global Developmental Delay HP:0001263, Intellectual disability HP:0001249, Impaired social interactions HP:0000735, Language impairment HP:0002463, Behavioral abnormality HP:0000708, Abnormality of the face HP:0000271 | Overlapping  Arachnoid cyst HP:0100702, Abnormal earlobe morphology HP:0000363, Pilonidal sinus HP:0010769, Velopharyngeal insufficiency HP:0000220 |
| 170 | ***NOTCH2*** | unknown (AD)/LP | Hernia HP:0100790, Weight loss HP:0001824, Synophrys HP:0000664 (mild) | ***ASXL1*** | unknown (AD)/LP | Intellectual disability, profound HP:0002187, Myoclonus HP:0001336, Neurodevelopmental delay HP:0012758, Seizure HP:0001250, Weight loss HP:0001824, Long face HP:0000276, Narrow palate HP:0000189 | Additive  Autistic behavior HP:0000729, Behavioral abnormality HP:0000708, Stereotypy HP:0000733, Pica HP:0011856, Language impairment HP:0002463, Agitation HP:0000713, Aggressive behavior HP:0000718, Polydipsia HP:0001959, Open mouth HP:0000194, Mandibular prognathia HP:0000303, Sloping forehead HP:0000340, Prominent nasal tip HP:0005274, Thin ear helix HP:0009905 |
| 77 | ***PTEN*** | *de novo* (AD)/LP | Intellectual disability, borderline HP:0006889, EEG abnormality HP:0002353 | ***PSMD12*** | *de novo* (AD)/VUS | Autistic behavior HP:0000729, Intellectual disability, borderline HP:0006889, EEG abnormality HP:0002353, | Overlapping  Long face HP:0000276, Deeply set eye HP:0000490 (mild), Abnormal palate morphology HP:0000174 (flat), Hyperextensibility at elbow HP:0010485 |
| 395 | ***RAB3GAP1*** | homozygous (AR)/P | Developmental delay HP:0001263, Congenital cataract HP:0000519, Deep-set eyes HP:0000490, Large ears HP:0000400, Postnatal failure to thrive HP:0001508, Hypotonia HP:0001290 | ***PAH*** | homozygous (AR)/LP | Developmental delay HP:0001263, Congenital cataract HP:0000519, Phenylketonuria HP:0002187 | Additive  Frontal bossing HP:0002007, Short palpebral fissures HP:0000589, Long philtrum HP:0000343 |
| 213 | ***SHANK3*** | *de novo* (AD)/P | Global Developmental Delay HP:0001263, Behavioral abnormality HP:0000708 | ***OFD1*** | maternal (XLR)/VUS | Global develpmental delay HP:0001263, High palate HP:0000218, Arachnodactyly HP:0001166, Adducted thumb HP:0001181 | Overlapping  Long face HP:0000276, Lumbar scoliosis HP:0004626, Thoracic kyphosis HP:0002942, Cubitus valgus HP:0002967, Finger joint hypermobility HP:0006094, Open mouth HP:0000194, Crohn's disease HP:0100280 |
| 105 | ***SOX11*** | *de novo* (AD)/LP | Neurodevelopmental delay HP:0012758 | ***DYNC1I2*** | Compound heterozygosity (AR)/VUS | Neurodevelopmental delay HP:0012758, Behavioral abnormality HP:0000708, Absent speech HP:0001344 | Overlapping  Cleft palate HP:0000175, Inguinal hernia HP:0000023 |
| 209 | ***TCF4*** | *de novo* (AD)/P | Intellectual disability HP:0001249, Behavioral abnormality HP:0000708, Delayed speech and language development HP:0000750, Absent speech HP:0001344, Broad-based gait HP:0002136, Pes valgus HP:0008081 | ***KMT2A*** | *de novo* (AD)/VUS | Intellectual disability HP:0001249, Behavioral abnormality HP:0000708, Delayed speech and language development HP:0000750, Absent speech HP:0001344, Broad-based gait HP:0002136 | Overlapping  Dysphagia HP:0002015, Hydrocephalus HP:0000238 |
| 375 | ***TLK2*** | *de novo* (AD)/LP | Global Developmental Delay HP:0001263, Scoliosis HP:0002650, EEG abnormality HP:0002353, High palate HP:0000218 | ***DYNC1H1*** | *de novo* (AD)/LP | Global Developmental Delay HP:0001263, Thin corpus callosum HP:0033725, EEG abnormality HP:0002353 | Overlapping  Polyphagia HP:0002591, Synophrys HP:0000664, Hypertelorism HP:0000316, Aplasia/Hypoplasia of the phalanges of the 5th finger HP:0009376, Macrocephaly HP:0000256, Exostoses HP:0100777 |

*Phenotypes derived from the clinical summary available at <https://www.omim.org/> and/or from the relevant literature (where indicated)

| Table S5. Genes published during the study | | | | | | | | |
| --- | --- | --- | --- | --- | --- | --- | --- | --- |
| Family | **Gene** | **NM_code** | **cDNA change** | **Protein change** | **Inheritance** | **ACMG classification** | **Gene (PMID)**^a^ | **Case published (PMID)** |
| 392 | *DDX53* | NM_182699.4 | c.834G>A | p.(Met278Ile) | maternal (XLR) | VUS | 38234782 | 38234782 |
| 366 | *DENND5B* | NM_144973.4 | c.1488C>A | p.(Tyr496*) | unknown (AD) | P | 38387458 | To be published |
| 47 | *GLRA2* | NM_002063.4 | c.1186C>A | p.(Pro396Thr) | maternal (XL) | VUS | 35294868 | 35294868 |
| 202 | *H3F3B* | NM_005324.5 | c.377A>G | p.(Gln126Arg) | *de novo* (AD) | P | 33268356 | 33268356 |
| 65 | *KCNK18* | NM_181840.1 | c.755C>T c.487T>G | p.(Ser252Leu) mat p.(Tyr163Asp) pat | compound heterozygote (AR) | LP | 34199759 | 34199759 |
| 122 | *RICTOR* | NM_152756.5 | c.214C>T | p.(His72Tyr) | *de novo* (AD) | LP | 39738822 | 39738822 |
| 150 | *RNF40* | NM_014771.4 | c.2320C>T | p.(Arg774Trp) | *de novo* (AD) | LP | To be published | To be published |
| 107 | *RPH3A* | NM_001143854.2 | c.1853A>G | p.(Asn618Ser) | *de novo* (AD) | LP | 37403762 | 37403762 |
| 318 | *SF3B3* | NM_012426.5 | c.647G>A | p.(Gly216Asp) | *de novo* (AD) | LP | To be published | To be published |
| 66 | *SMARCA1* | NM_001282874.2 | c.566G>A | p.(Arg189Gln) | maternal (XLR) | VUS | To be published | To be published |
| 382 | *SUPT5H* | NM_001111020.3 | c.320-1G>A | p.? | *de novo* (AD) | P | To be published | To be published |
| 376 | *UPF1* | NM_002911.4 | c.2495G>A | p.(Arg832His) | *de novo* (AD) | LP | To be published | To be published |
| 439 | *ZMYM3* | NM_201599.3 | c.905G>A | p.(Arg302His) | maternal (XLR) | VUS | 36586412 | 36586412 |

^a^ Manuscripts reporting the association between gene and disease.

**REFERENCES**

Bosch E, Güse E, Kirchner P, Winterpacht A, Walther M, Alders M, Kerkhof J, Ekici AB, Sticht H, Sadikovic B, Reis A, Vasileiou G (2024) The missing link: ARID1B non-truncating variants causing Coffin-Siris syndrome due to protein aggregation. Hum Genet 143: 965-978. doi: 10.1007/s00439-024-02688-9

Consortium ASDWGoTPG (2017) Meta-analysis of GWAS of over 16,000 individuals with autism spectrum disorder highlights a novel locus at 10q24.32 and a significant overlap with schizophrenia. Mol Autism 8: 21. doi: 10.1186/s13229-017-0137-9

De Rubeis S, He X, Goldberg AP, Poultney CS, Samocha K, Cicek AE, Kou Y, Liu L, Fromer M, Walker S, Singh T, Klei L, Kosmicki J, Shih-Chen F, Aleksic B, Biscaldi M, Bolton PF, Brownfeld JM, Cai J, Campbell NG, Carracedo A, Chahrour MH, Chiocchetti AG, Coon H, Crawford EL, Curran SR, Dawson G, Duketis E, Fernandez BA, Gallagher L, Geller E, Guter SJ, Hill RS, Ionita-Laza J, Jimenz Gonzalez P, Kilpinen H, Klauck SM, Kolevzon A, Lee I, Lei I, Lei J, Lehtimaki T, Lin CF, Ma'ayan A, Marshall CR, McInnes AL, Neale B, Owen MJ, Ozaki N, Parellada M, Parr JR, Purcell S, Puura K, Rajagopalan D, Rehnstrom K, Reichenberg A, Sabo A, Sachse M, Sanders SJ, Schafer C, Schulte-Ruther M, Skuse D, Stevens C, Szatmari P, Tammimies K, Valladares O, Voran A, Li-San W, Weiss LA, Willsey AJ, Yu TW, Yuen RK, Study DDD, Homozygosity Mapping Collaborative for A, Consortium UK, Cook EH, Freitag CM, Gill M, Hultman CM, Lehner T, Palotie A, Schellenberg GD, Sklar P, State MW, Sutcliffe JS, Walsh CA, Scherer SW, Zwick ME, Barett JC, Cutler DJ, Roeder K, Devlin B, Daly MJ, Buxbaum JD (2014) Synaptic, transcriptional and chromatin genes disrupted in autism. Nature 515: 209-15. doi: 10.1038/nature13772

Fu JM, Satterstrom FK, Peng M, Brand H, Collins RL, Dong S, Wamsley B, Klei L, Wang L, Hao SP, Stevens CR, Cusick C, Babadi M, Banks E, Collins B, Dodge S, Gabriel SB, Gauthier L, Lee SK, Liang L, Ljungdahl A, Mahjani B, Sloofman L, Smirnov AN, Barbosa M, Betancur C, Brusco A, Chung BHY, Cook EH, Cuccaro ML, Domenici E, Ferrero GB, Gargus JJ, Herman GE, Hertz-Picciotto I, Maciel P, Manoach DS, Passos-Bueno MR, Persico AM, Renieri A, Sutcliffe JS, Tassone F, Trabetti E, Campos G, Cardaropoli S, Carli D, Chan MCY, Fallerini C, Giorgio E, Girardi AC, Hansen-Kiss E, Lee SL, Lintas C, Ludena Y, Nguyen R, Pavinato L, Pericak-Vance M, Pessah IN, Schmidt RJ, Smith M, Costa CIS, Trajkova S, Wang JYT, Yu MHC, Cutler DJ, De Rubeis S, Buxbaum JD, Daly MJ, Devlin B, Roeder K, Sanders SJ, Talkowski ME, (ASC) ASC, (Broad-CCDG) BICfCDG, Consortium i-B (2022) Rare coding variation provides insight into the genetic architecture and phenotypic context of autism. Nat Genet 54: 1320-1331. doi: 10.1038/s41588-022-01104-0

Geetha TS, Michealraj KA, Kabra M, Kaur G, Juyal RC, Thelma BK (2014) Targeted deep resequencing identifies MID2 mutation for X-linked intellectual disability with varied disease severity in a large kindred from India. Hum Mutat 35: 41-4. doi: 10.1002/humu.22453

Gumus E (2020) Extending the phenotype of Xia-Gibbs syndrome in a two-year-old patient with craniosynostosis with a novel de novo AHDC1 missense mutation. Eur J Med Genet 63: 103637. doi: 10.1016/j.ejmg.2019.03.001

Guo H, Wang T, Wu H, Long M, Coe BP, Li H, Xun G, Ou J, Chen B, Duan G, Bai T, Zhao N, Shen Y, Li Y, Wang Y, Zhang Y, Baker C, Liu Y, Pang N, Huang L, Han L, Jia X, Liu C, Ni H, Yang X, Xia L, Chen J, Shen L, Zhao R, Zhao W, Peng J, Pan Q, Long Z, Su W, Tan J, Du X, Ke X, Yao M, Hu Z, Zou X, Zhao J, Bernier RA, Eichler EE, Xia K (2018) Inherited and multiple de novo mutations in autism/developmental delay risk genes suggest a multifactorial model. Mol Autism 9: 64. doi: 10.1186/s13229-018-0247-z

Højland AT, Lolas I, Okkels H, Lautrup CK, Diness BR, Petersen MB, Nielsen IK (2018) First reported adult patient with TARP syndrome: A case report. Am J Med Genet A 176: 2915-2918. doi: 10.1002/ajmg.a.40638

Iossifov I, O'Roak BJ, Sanders SJ, Ronemus M, Krumm N, Levy D, Stessman HA, Witherspoon KT, Vives L, Patterson KE, Smith JD, Paeper B, Nickerson DA, Dea J, Dong S, Gonzalez LE, Mandell JD, Mane SM, Murtha MT, Sullivan CA, Walker MF, Waqar Z, Wei L, Willsey AJ, Yamrom B, Lee YH, Grabowska E, Dalkic E, Wang Z, Marks S, Andrews P, Leotta A, Kendall J, Hakker I, Rosenbaum J, Ma B, Rodgers L, Troge J, Narzisi G, Yoon S, Schatz MC, Ye K, McCombie WR, Shendure J, Eichler EE, State MW, Wigler M (2014) The contribution of de novo coding mutations to autism spectrum disorder. Nature 515: 216-21. doi: 10.1038/nature13908

Jaganathan K, Kyriazopoulou Panagiotopoulou S, McRae JF, Darbandi SF, Knowles D, Li YI, Kosmicki JA, Arbelaez J, Cui W, Schwartz GB, Chow ED, Kanterakis E, Gao H, Kia A, Batzoglou S, Sanders SJ, Farh KK (2019) Predicting Splicing from Primary Sequence with Deep Learning. Cell 176: 535-548.e24. doi: 10.1016/j.cell.2018.12.015

Johnston JJ, Teer JK, Cherukuri PF, Hansen NF, Loftus SK, Chong K, Mullikin JC, Biesecker LG, (NISC) NISC (2010) Massively parallel sequencing of exons on the X chromosome identifies RBM10 as the gene that causes a syndromic form of cleft palate. Am J Hum Genet 86: 743-8. doi: 10.1016/j.ajhg.2010.04.007

Khayat MM, Hu J, Jiang Y, Li H, Chander V, Dawood M, Hansen AW, Li S, Friedman J, Cross L, Bijlsma EK, Ruivenkamp CAL, Sansbury FH, Innis JW, O'Shea JO, Meng Q, Rosenfeld JA, McWalter K, Wangler MF, Lupski JR, Posey JE, Murdock D, Gibbs RA (2021) missense mutations in Xia-Gibbs syndrome. HGG Adv 2. doi: 10.1016/j.xhgg.2021.100049

Lim CS, Kim MJ, Choi JE, Islam MA, Lee YK, Xiong Y, Shim KW, Yang JE, Lee RU, Lee J, Park P, Kwak JH, Seo H, Kim CH, Lee JH, Lee YS, Hwang SK, Lee K, Lee JA, Kaang BK (2021) Dysfunction of NMDA receptors in neuronal models of an autism spectrum disorder patient with a DSCAM mutation and in Dscam-knockout mice. Mol Psychiatry 26: 7538-7549. doi: 10.1038/s41380-021-01216-9

Maleki MH, Jalilian FA, Khayat H, Mohammadi M, Pourahmad F, Asadollahi K, Pakzad I, Sadeghifard N, Soroush S, Emaneini M, Taherikalani M (2014) Detection of highly ciprofloxacin resistance acinetobacter baumannii isolated from patients with burn wound infections in presence and absence of efflux pump inhibitor. Maedica (Buchar) 9: 162-7.

Mermet-Meillon F, Mercan S, Bauer-Probst B, Allard C, Bleu M, Calkins K, Knehr J, Altorfer M, Naumann U, Sprouffske K, Barys L, Sesterhenn F, Galli GG (2024) Protein destabilization underlies pathogenic missense mutations in ARID1B. Nat Struct Mol Biol 31: 1018-1022. doi: 10.1038/s41594-024-01229-2

Nasvytis M, Čiauškaitė J, Jurkevičienė G (2024) GNB1 Encephalopathy: Clinical Case Report and Literature Review. Medicina (Kaunas) 60. doi: 10.3390/medicina60040589

Niceta M, Barresi S, Pantaleoni F, Capolino R, Dentici ML, Ciolfi A, Pizzi S, Bartuli A, Dallapiccola B, Tartaglia M, Digilio MC (2019) TARP syndrome: Long-term survival, anatomic patterns of congenital heart defects, differential diagnosis and pathogenetic considerations. Eur J Med Genet 62: 103534. doi: 10.1016/j.ejmg.2018.09.001

Oladnabi M, Musante L, Larti F, Hu H, Abedini SS, Wienker T, Ropers HH, Kahrizi K, Najmabadi H (2015) New evidence for the role of calpain 10 in autosomal recessive intellectual disability: identification of two novel nonsense variants by exome sequencing in Iranian families. Arch Iran Med 18: 179-84.

Trajkova S, Kerkhof J, Rossi Sebastiano M, Pavinato L, Ferrero E, Giovenino C, Carli D, Di Gregorio E, Marinoni R, Mandrile G, Palermo F, Carestiato S, Cardaropoli S, Pullano V, Rinninella A, Giorgio E, Pippucci T, Dimartino P, Rzasa J, Rooney K, McConkey H, Petlichkovski A, Pasini B, Sukarova-Angelovska E, Campbell CM, Metcalfe K, Jenkinson S, Banka S, Mussa A, Ferrero GB, Sadikovic B, Brusco A (2024) DNA methylation analysis in patients with neurodevelopmental disorders improves variant interpretation and reveals complexity. HGG Adv 5: 100309. doi: 10.1016/j.xhgg.2024.100309

Wang J, Al-Ouran R, Hu Y, Kim SY, Wan YW, Wangler MF, Yamamoto S, Chao HT, Comjean A, Mohr SE, Perrimon N, Liu Z, Bellen HJ, UDN (2017) MARRVEL: Integration of Human and Model Organism Genetic Resources to Facilitate Functional Annotation of the Human Genome. Am J Hum Genet 100: 843-853. doi: 10.1016/j.ajhg.2017.04.010

Wang T, Guo H, Xiong B, Stessman HA, Wu H, Coe BP, Turner TN, Liu Y, Zhao W, Hoekzema K, Vives L, Xia L, Tang M, Ou J, Chen B, Shen Y, Xun G, Long M, Lin J, Kronenberg ZN, Peng Y, Bai T, Li H, Ke X, Hu Z, Zhao J, Zou X, Xia K, Eichler EE (2016) De novo genic mutations among a Chinese autism spectrum disorder cohort. Nat Commun 7: 13316. doi: 10.1038/ncomms13316

Wiel L, Baakman C, Gilissen D, Veltman JA, Vriend G, Gilissen C (2019) MetaDome: Pathogenicity analysis of genetic variants through aggregation of homologous human protein domains. Hum Mutat 40: 1030-1038. doi: 10.1002/humu.23798

Zeng T, Li YI (2022) Predicting RNA splicing from DNA sequence using Pangolin. Genome Biol 23: 103. doi: 10.1186/s13059-022-02664-4
